# Dioxin(-like)-Related Biological Effects through Integrated Chemical-wide and Metabolome-wide Analyses

**DOI:** 10.1021/acs.est.3c07588

**Published:** 2023-12-27

**Authors:** Yujia Zhao, Jeroen Meijer, Douglas I. Walker, Juni Kim, Lützen Portengen, Dean P. Jones, Fatemeh Saberi Hosnijeh, Jelle Vlaanderen, Roel Vermeulen

**Affiliations:** † Institute for Risk Assessment Sciences, Utrecht University, Utrecht 3584 CM, The Netherlands; ‡ Department Environment & Health, Vrije Universiteit, Amsterdam 1081 HV, The Netherlands; § Gangarosa Department of Environmental Health, Rollins School of Public Health, Emory University, Atlanta, Georgia 30322, United States; ∥ Division of Pulmonary, Allergy, Critical Care and Sleep Medicine, School of Medicine, Emory University, Atlanta, Georgia 30322, United States; ⊥ Julius Center for Health Sciences and Primary Care, University Medical Centre Utrecht, Utrecht 3584 CX, The Netherlands

**Keywords:** dioxin(-like) exposures, chemical-wide association study, metabolome-wide association study, occupational population, biological pathways, exposome

## Abstract

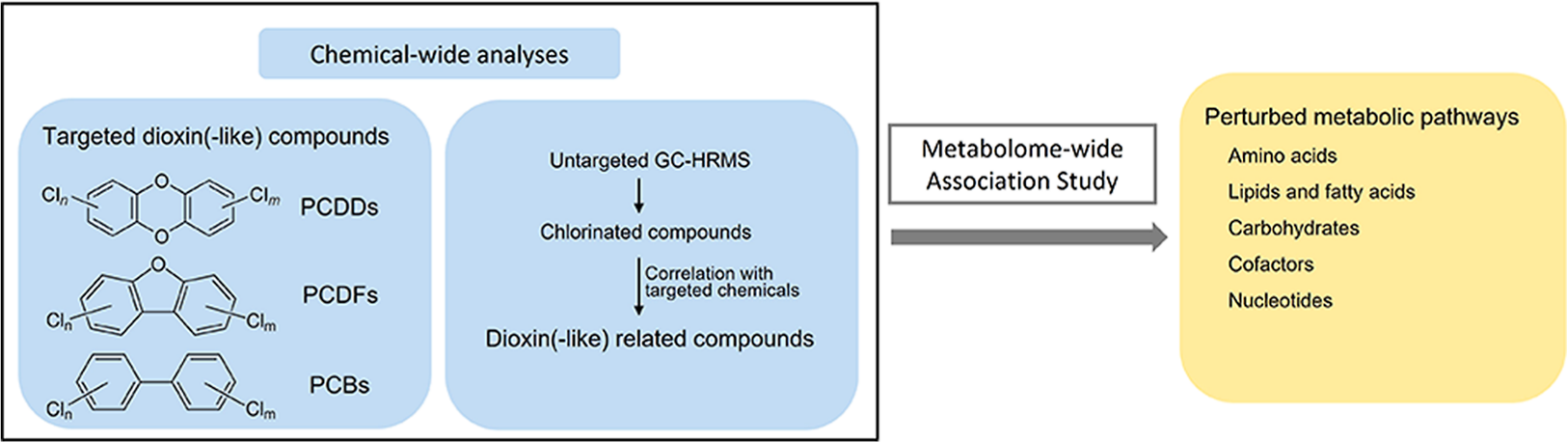

Dioxin(-like) exposures are linked to adverse health effects, including cancer. However, metabolic alterations induced by these chemicals remain largely unknown. Beyond known dioxin(-like) compounds, we leveraged a chemical-wide approach to assess chlorinated co-exposures and parent compound products [termed dioxin(-like)-related compounds] among 137 occupational workers. Endogenous metabolites were profiled by untargeted metabolomics, namely, reversed-phase chromatography with negative electrospray ionization (C18-negative) and hydrophilic interaction liquid chromatography with positive electrospray ionization (HILIC-positive). We performed a metabolome-wide association study to select dioxin(-like) associated metabolic features using a 20% false discovery rate threshold. Metabolic features were then characterized by pathway enrichment analyses. There are no significant features associated with polychlorinated dibenzo-*p*-dioxins (PCDDs), a subgroup of known dioxin(-like) compounds. However, 3,110 C18-negative and 2,894 HILIC-positive features were associated with at least one of the PCDD-related compounds. Abundant metabolic changes were also observed for polychlorinated dibenzofuran-related and polychlorinated biphenyl-related compounds. These metabolic features were primarily enriched in pathways of amino acids, lipid and fatty acids, carbohydrates, cofactors, and nucleotides. Our study highlights the potential of chemical-wide analysis for comprehensive exposure assessment beyond targeted chemicals. Coupled with advanced endogenous metabolomics, this approach allows for an in-depth exploration of metabolic alterations induced by environmental chemicals.

## Abstract 2

This study practices a novel exposure assessment of environmental chemicals, exemplified by dioxin(-like) compounds. This approach extends its scope beyond known chemicals to incorporate co-exposures and parental chemical metabolites and further investigate the biological implications of dioxin(-like) exposures.

## 1 Introduction

Dioxin(-like) compounds rank among the most notorious anthropogenic environmental toxicants and have been extensively studied over the past four decades.^1^ This chemical category includes three structurally related subclasses: polychlorinated dibenzo-*p*-dioxins (PCDDs), dioxin-like polychlorinated dibenzofurans (PCDFs), and dioxin-like polychlorinated biphenyls (PCBs).^2^ The risk assessment of dioxin(-like) compounds, like many other exposures, primarily focuses on individual chemicals, particularly the most toxic chemical, 2,3,7,8-tetrachlorodibenzo-*p*-dioxin (TCDD). TCDD is classified as a “known human carcinogen” and is associated with an increased risk of all cancers combined.^3^ Furthermore, TCDD has been implicated in toxicities concerning the immune, nervous, endocrine, and reproductive systems.^4^

A one-by-one assessment of the biological impact of chemicals may overlook important biological perturbations. In a metabolome-wide association study (MWAS) by Walker et al. on trichloroethylene (TCE), it was shown that most of the observed biological effects were associated stronger with unknown metabolic products of TCE, as opposed to TCE itself or prior known metabolites.^5^ This challenges the conventional practice of assessing chemical toxicity by focusing solely on parent compounds and known metabolites.^6^ An alternative strategy could be to first comprehensively map exposures to known compounds, co-exposures (e.g., unrecognized chemicals with analogous properties), and their metabolites, followed by associating these with biological changes. This integrated chemical-wide and metabolome-wide approach could yield a more comprehensive evaluation of biological effects.

**Figure 1:**
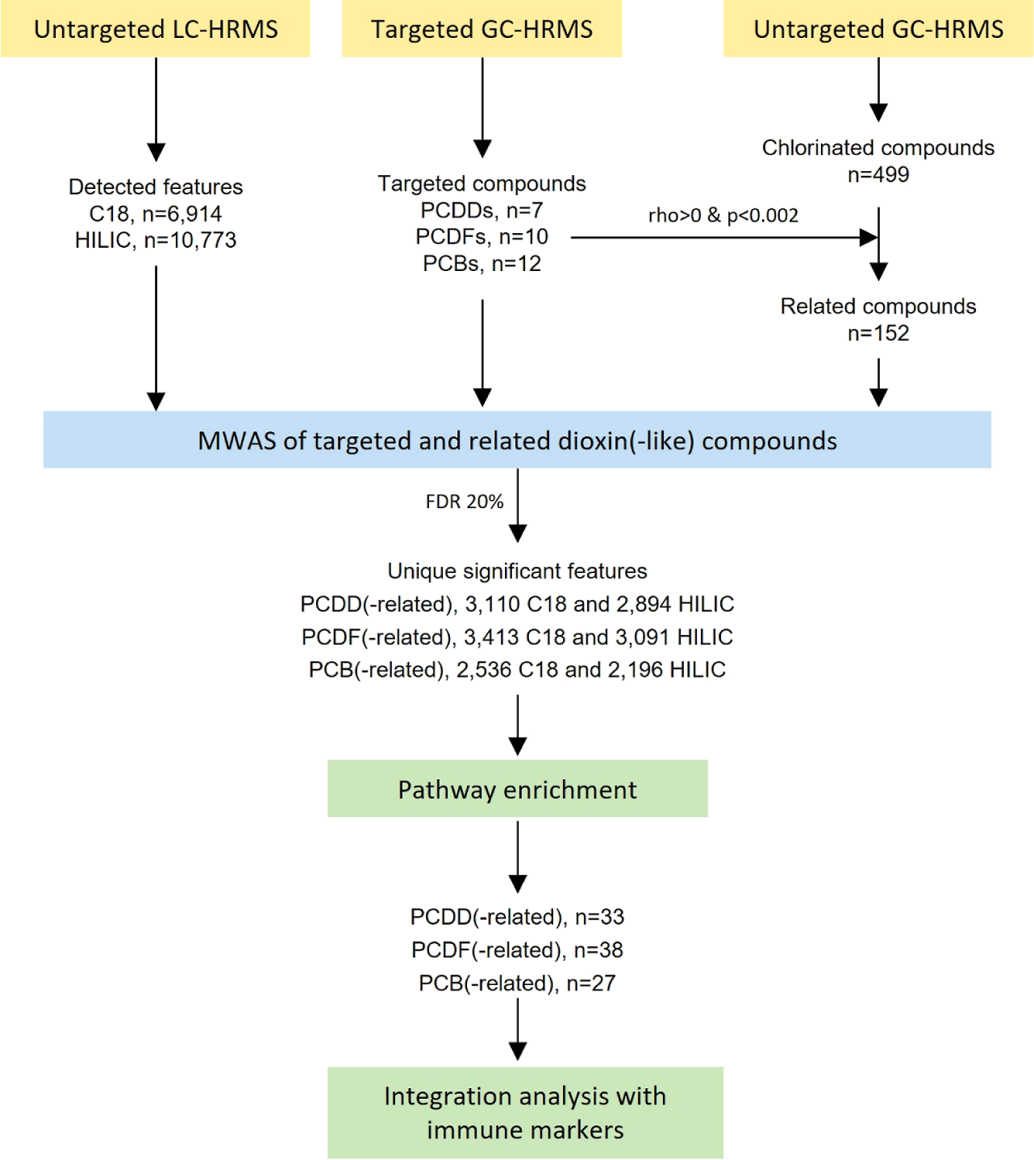
Workflow of the chemical-wide and metabolome-wide association analyses. Abbreviation: LC-HRMS, liquid chromatography with orbitrap high-resolution mass spectrometry; GC-HRMS, gas chromatography with high-resolution mass spectrometry; C18, C18-negative mode; HILIC, HILIC-positive mode; MWAS, metabolome-wide association study; and FDR, false discovery rate.

We illustrate here an example of a chemical-wide and metabolome-wide investigation (Figure 1) through (i) an exhaustive targeted analysis of dioxin(-like) compounds, (ii) connecting these targeted dioxin(-like) compounds to associated chlorinated compounds characterized using untargeted gas chromatography with high-resolution mass spectrometry (GC-HRMS), thus encompassing a thorough representation of dioxin(-like) exposures, and (iii) linking targeted and related dioxin(-like) compounds with biological changes assessed through metabolomics and targeted immunological phenotyping.

For these research goals, we used a highly unique subpopulation of the Dutch herbicide cohort, recognized as one of the most informative epidemiological studies in dioxin research.^7^ The cohort comprised workers of two chlorophenoxy herbicide-producing factories.^8,9^ One factory (factory A) experienced high TCDD exposure due to a reactor vessel explosion in 1963. Even decades later, TCDD levels in the blood of ex-factory A workers remained substantially higher than in the general population (4 ppt vs below the detection limit). Likewise, levels of dioxin-like PCDFs and PCBs generally exceeded background levels, as reported in monitoring data (Supporting Information, Table S1). This occupational cohort provides a distinctive opportunity to investigate the health and biological effects associated with dioxin(-like) exposures.

## 2 Methods

### 2.1 Study Population

The subjects involved in this study were drawn from the Dutch herbicide cohort. Details have been described elsewhere.^8−10^ Briefly, this cohort comprised workers from two factories (denoted as factories A and B) engaged in manufacturing chlorophenoxy herbicides during the 1950s–1980s in The Netherlands. Factory A’s primary products were 2,4,5-trichlorophenoxyacetic acid (2,4,5-T) and 2,4,5-trichlorophenol (2,4,5-TCP), which included potential contamination with TCDD and other PCDDs. In March 1963, an explosion occurred within an autoclave for the synthesis of 2,4,5-TCP, releasing its contents, including TCDD. Individuals working in production departments at factory A or present during the accident and subsequent cleanup were exposed to high levels of TCDD. Conversely, factory B produced 4-chloro-2-methylphenoxyacetic acid (MCPA), 4-chloro-2-methylphenoxy propanoic acid (MCPP), and, in smaller amounts, 2,4-dichlorophenoxyacetic acid (2,4-D). While potential by-products in factory B included PCDDs (mainly with 2 to 3 chlorine atoms) and dioxin-like PCDFs and PCBs, the presence of TCDD was unlikely.

Workers ever employed in factory A (*n* = 1167) and factory B (*n* = 1143) were enrolled in the cohort. During the third follow-up period (2007–2008), participants were selected for blood collection based on a stratified sampling strategy that considered their exposure status to chlorophenoxy herbicides, chlorophenols, and associated contaminants. The study enrolled 82 workers from factory A, half of whom had worked within production departments or participated in accident-related cleanup, alongside a randomly selected sample of 70 workers from factory B. All study subjects were male and completed a questionnaire covering basic information, anthropometric parameters (height and weight), and lifestyle habits (smoking status and alcohol consumption). Plasma was separated and stored at −80 °C.

### 2.2 Exposure Assessment for Dioxin(-like) Compounds

#### 2.2.1 Measurement of Targeted Dioxin(-like) Compounds

Previously identified dioxin(-like) compounds in plasma were quantified by targeted GC-HRMS in the Centers for Disease Control and Prevention, USA. Targeted dioxin(-like) compounds covered seven PCDDs (TCDD, 12378D, 123478D, 123678D, 123789D, 1234678D, OCDD), ten dioxin-like PCDFs (2378F, 12378F, 23478F, 123478F, 123678F, 123789F, 234678F, 1234678F, 1234789F, OCDF), and 12 dioxin-like PCBs (PCB77, PCB81, PCB126, PCB169, PCB105, PCB114, PCB118, PCB123, PCB156, PCB157, PCB167, PCB189) (Table S1). Concentrations of these targeted dioxin(-like) compounds were adjusted for total lipids and reported as parts per trillion (ppt). Values below detection limits were imputed using the maximum likelihood method.^11^

Given TCDD’s protracted half-life in humans, its presence in biofluids persists for decades post initial exposure. We previously developed a predictive model to back-extrapolate plasma TCDD levels at the time of the last exposure (TCDD_max_). This prediction integrated measured TCDD levels within a one-compartment first-order kinetic model, where TCDD’s half-life was established at 7.1 years (*t*_1/2_)^12^
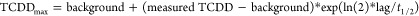


Lag periods for factory A workers were determined by their occupational history and involvement in the cleanup, following the 1963 accident.^12^ Factory B workers were not assigned a lag period; the measured TCDD levels were taken as the TCDD_max_. The average TCDD concentration detected in factory B served as the background level in the model.

#### 2.2.2 Measurement of Dioxin(-like)-Related Compounds

We characterized all possible dioxin(-like) exposures using untargeted GC-HRMS in the Rollins School of Public Health, Emory University, USA. Plasma samples were prepared and analyzed using methods described previously.^13^ Plasma samples were extracted using 4:1 hexane/ethyl acetate and analyzed in duplicate using a Thermo Scientific 1310 GC connected to a Thermo Scientific Q Exactive GC Orbitrap GC–MS/MS. The GC-HRMS was operated in full-scan mode over a mass-to-charge (*m*/*z*) range of 85–850 and 60,000 resolutions. Uniquely detected metabolic features consisting of *m*/*z*, retention time, and ion abundance were extracted and aligned using extensible computational mass spectrometry (XCMS) software.^14^ To identify unique mass spectra, we performed a data-driven clustering algorithm using *RamClustR*,^15^ which aggregates feature intensities based on correlation and retention-time grouping and provides a weight-averaged intensity for each group of features corresponding to an individual compound. After *m*/*z* clustering, 11,004 unique mass spectra, referred to as chemical features, were identified from the untargeted GC-HRMS data.

To identify additional compounds related to targeted dioxin(-like) compounds, mass spectra corresponding to each chemical feature were evaluated for chlorinated isotopic patterns by linking monoisotopic masses to M + 2, M + 4, M + 6, and M + 8 isotopic envelopes using the R package *nontarget*.^16^ Compounds showing a significant positive correlation (Spearman’s rank correlation coefficient >0 with a *p*-value below 0.002; corresponding to a 20% false discovery threshold) with any of the 29 targeted dioxin(-like) compounds were designated as dioxin(-like)-related compounds. This criterion was chosen to mitigate the impact of false positives from multiple tests (Text S1). The relationships among targeted dioxin(-like) compounds and those identified as dioxin(-like) related were depicted by a correlation-based network. Node clustering was identified using a multilevel community detection algorithm implemented in the *igraph* package.^17^ Finally, the network and clustering were visualized using Cytoscape software.^18^

Throughout this paper, chlorinated compounds that were correlated with at least one targeted PCDD are called “PCDD-related compounds”. Similarly, chlorinated compounds correlated with at least one PCDF or PCB are called “PCDF-related compounds” or “PCB-related compounds”, respectively.

### 2.3 High-Resolution Metabolomics

Untargeted metabolomic profiling in plasma was conducted using LC-HRMS (Dionex Ultimate 3000, Q-Exactive HF, Thermo Scientific) in Emory University, as previously described.^19^ Two complementary LC columns were used to maximize coverage, including reversed-phase with negative electrospray ionization (C18-negative) and HILIC-positive.^20^ Plasma samples were processed by adding two volumes of acetonitrile to precipitate proteins, and triplicate analyses were conducted in each mode. The HRMS was operated in full scan mode at 120,000 resolution over a *m*/*z* range 85–1275. Raw data files were extracted and aligned using *apLCMS*,^21^ with modifications by *xMSanalyzer*.^22^ In total, 10,477 and 16,605 metabolite features were detected for C18-negative and HILIC-positive mode, respectively. Before data analysis, metabolite features were batch-corrected using *ComBat*(23) and averaged, followed by removing features with a coefficient of variation among technical replicates ≥100% and detected in <60% of the study subjects. The remaining missing values were imputed using a left-censored quantile regression approach, implemented in *imputeLCMD*.^24^ After imputation, 6,914 C18-negative and 10,773 HILIC-positive LC-HRMS features were retained for subsequent analyses.

### 2.4 MWAS of Dioxin(-like) Exposures

Targeted and related dioxin(-like) compounds and metabolic features were naturally log-transformed for analyses. In the MWAS, we used the linear regression framework as implemented in the *Omics* R package,^25^ by regressing metabolic features one-by-one on a specific exposure compound [either a known or related dioxin(-like) compound]. These models were adjusted for age (continuous variable), factory (categorical variable), and body mass index (BMI; kg/m^2^, continuous variable) (model 1). To account for multiple comparisons, the Benjamini–Hochberg (BH) procedure^26^ was applied for the MWAS of each exposure, and a false discovery rate (FDR) threshold of 20% was adopted to identify metabolite features associated with the exposure. Separate FDR procedures were applied to metabolic features detected by the C18-negative and HILIC-positive modes.

In this study, dioxin(-like) exposures primarily originated from occupational activities and were minimally associated with lifestyle factors such as smoking and alcohol consumption. Moreover, we conducted an expanded analysis to incorporate smoking status and alcohol intake as additional covariates (model 2). However, corresponding exposure-feature coefficients in model 2 exhibited minimal deviations, less than 3% on average, compared to those in model 1 (data not shown). Consequently, smoking and alcohol consumption were less likely to act as confounders in this study, leading us to select model 1 for subsequent analyses.

We categorized metabolic features into three subclasses based on their associations with PCDD(-related) compounds, PCDF(-related) compounds, and PCB(-related) compounds under a FDR 20%. Subsequently, separate pathway enrichments were performed for each subclass of metabolic features.

### 2.5 Biological Pathway Enrichment and Metabolite Annotation

To characterize metabolic features associated with dioxin(-like) exposures, we first matched the significant features to an internal compound database, confirmed by authentic reference standards, denoting level 1 confidence.^27^ Features without matches with authentic standards were annotated using *xMSannotator*.^28^ This tool categorizes annotations into different confidence tiers using a multistage clustering algorithm based on database matches. We searched against the Human Metabolome Database (HMDB)^29^ with a mass tolerance of ±5 ppm and a retention time tolerance of ±5 s. The adducts were “M + H”, “M + 2H”, “M + ACH + 2H”, “M + Na”, “M + ACN + H”, “M + ACN + Na”, “2M + H”, and “M + H + H_2_O” for positive mode and “M – H”, “M – H_2_O – H”, “M + Na – 2H″, “M + Cl”, “M + Hac – H”, and “2M – H” for negative mode. Annotations suggested with a high confidence level by *xMSannotator* were presented as level 4 confidence annotations, according to Schymanski et al.^27^

Metabolic pathways associated with dioxin(-like) exposures were identified using Mummichog (version 1.0.10) with a mass tolerance of ±5 ppm.^30^ Associated metabolic pathways were identified using a pathway significance threshold <0.05 as well as the presence of at least four metabolites associated with the exposures.

### 2.6 Integration of Metabolic Pathways and Immune Phenotypic Measures

To explore the potential mechanisms underlying dioxin(-like) exposure toxicity, we used a network-based integration approach to evaluate the relationship between the metabolic pathways associated with dioxin(-like) exposures and immune phenotypic end points. Immune markers, including cytokines and growth factors (*n* = 21), hematologic parameters (i.e., cell counts) (*n* = 23), humoral immunity markers [immunoglobulins (Ig) and complement factors (C)] (*n* = 7), and lymphoma makers (*n* = 3), were previously measured for factory A workers^31−34^ (Table S3).

For factory A workers, principal component analysis (PCA) was conducted on the intensities of significant metabolic features within each enriched Mummichog-identified pathway. Subsequently, first principle component (PC1) scores were computed as a summary measure for each respective pathway. These PC1 scores, representing metabolic pathways, were subjected to partial least-squares (PLS) regression via the *xMWAS* package^35^ to explore potential associations with all immune markers. In PLS regression, the association score between variables from two matrices approximates their correlation coefficient, determined by PLS components and regression coefficients.^36^ The resultant pairwise associations, marked by an |association score| exceeding 0.3 and *p*-value below 0.05, were used to build a network to visualize the connections between the pathways and biomarkers. A multilevel community detection method was applied to uncover clusters of pathways and biomarkers.^37^ The network and identified communities were visualized using Cytoscape.^18^

## 3 Results

### 3.1 Study Population and Dioxin(-like) Exposures

After the inclusion of workers with a diagnosis of cancer (except for skin cancer) (6 from factory A and 9 from factory B), 76 workers from factory A and 61 workers from factory B were retained in the analyses. Workers in factory A were older compared to those in factory B (69.0 vs 58.8 years, *p* < 0.001) (Table 1). No significant differences were observed in BMI, alcohol intake, and smoking status between the two factories.

**Table 1:** Characteristics of Study Participants^a^

	factory A (*n* = 76)	factory B (*n* = 61)	*p*-value^b^
age (years), mean (SD)	69.0 (7.7)	58.8 (9.0)	<0.001
body mass index (kg/m^2^)^c^, mean (SD)	26.9 (3.0)	27.1 (3.6)	0.726
alcohol intake (units/week), mean (SD)	13.2 (13.6)	13.7 (15.1)	0.835
smoking status, *n* (%)			0.594
never smokers	12 (15.8%)	13 (21.3%)	
former smokers	46 (60.5%)	32 (52.5%)	
current smokers	18 (23.7%)	16 (26.2%)	

a Abbreviations: SD, standard deviation.

b *p* values from the *t*-test for continuous variables and the chi-square test for categorical variables, subjects from factory A vs factory B.

c Body mass index (BMI) was calculated as the weight in kilograms divided by the square of the height in meters.

As expected, the concentrations of PCDDs were markedly higher in factory A workers compared to factory B workers (all *p* < 0.05) (Table S1). Notably, the difference in TCDD levels between the two factories was substantial (median 4.35 vs 0.30 ppt, *p* < 0.0001). Levels of dioxin-like PCDFs and PCBs were comparable across both factories. We observed moderate correlations among PCDDs and some high correlations among PCBs (*r*_s_ > 0.9), while most PCDFs were only weakly correlated (Figure S3).

**Figure 2:**
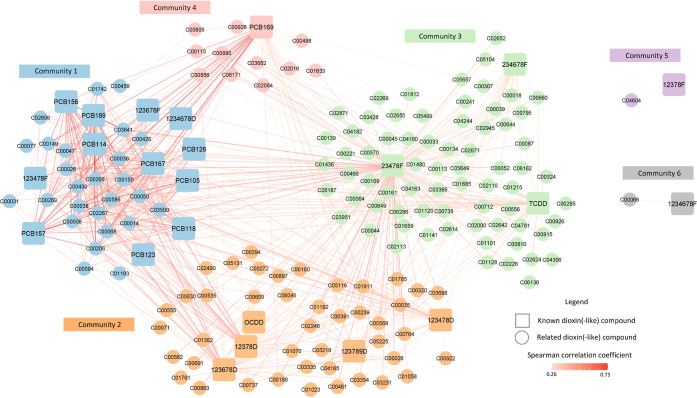
Network of targeted and related dioxin(-like) compounds network was generated based upon Spearman correlations between targeted dioxin(-like) compounds and their related compounds. Community detection was used to identify closely related nodes, which were indicated by node color. Correlation magnitude was indicated by the edge color.

Of the 499 suspected chlorinated compounds detected by untargeted GC-HRMS, 152 were identified as possible dioxin(-like)-related compounds. Specifically, 109 chlorinated compounds correlated to at least one PCDD, 136 to at least one PCDF, and 58 to at least one PCB (Table S4). Because of the correlated properties of targeted compounds, the overlap among the three categories of dioxin(-like)-related compounds was considerable (Figure S4). Our analysis of the network integrating targeted and related dioxin(-like) compounds identified six densely interconnected communities (Figure 2 and Table S5). All PCBs except PCB169 were grouped in Community 1, while five PCDDs were in Community 2. TCDD was clustered with 23478F and 234678F in Community 3.

### 3.2 MWAS of Dioxin(-like) Exposures

The numbers of metabolic features significantly associated with each targeted and its related dioxin(-like) compounds at a 20% FDR threshold are given in Table 2. While no feature was significantly associated with any targeted PCDD, we found that the PCDD-related compounds contributed substantially to metabolic alterations. Specifically, 3,110 C18-negative and 2,894 HILIC-positive features were found to be associated with at least one of the PCDD-related compounds. This phenomenon of enriched metabolic changes is held for PCDFs and PCBs, alongside their corresponding-related compounds. Predicted maximum levels of TCDD were not associated with any metabolic feature (Figure S5).

**Table 2:** Number of Metabolic Features Significantly Associated with Dioxin(-like) Exposures^a^

congener	C18-negative features^b^			HILIC-positive features^b^		
	targeted compound	related compounds^c^	total^d^	targeted compound	related compounds^c^	total^d^
PCDDs						
TCDD	0	2528	2528	0	776	776
12378D	0	1789	1789	0	998	998
123478D	0	2147	2147	0	2163	2163
123678D	0	2058	2058	0	1099	1099
123789D	0	1010	1010	0	1604	1604
1234678D	0	125	125	0	46	46
OCDD	0	413	413	0	158	158
total^d^	0	3110	3110	0	2894	2894
dioxin-like PCDFs						
2378F	2	NA	2	5	NA	5
12378F	0	267	267	0	3	3
23478F	0	3240	3240	0	2839	2839
123478F	0	1572	1572	0	682	682
123678F	1	902	903	0	156	156
123789F	0	0	0	0	0	0
234678F	0	1688	1688	1	727	727
1234678F	0	4	4	0	302	302
1234789F	0	NA	0	0	NA	0
OCDF	1	NA	1	1	NA	1
total^d^	4	3411	3413	7	3086	3091
dioxin-like PCBs						
PCB77	0	NA	0	143	NA	143
PCB81	0	NA	0	0	NA	0
PCB126	237	503	692	42	77	112
PCB169	0	2153	2153	0	2053	2053
PCB105	151	1182	1277	12	124	127
PCB114	0	1314	1314	0	740	740
PCB118	319	1182	1360	6	124	126
PCB123	100	292	360	9	67	71
PCB156	0	1270	1270	0	182	182
PCB157	0	1270	1270	0	182	182
PCB167	346	1540	1662	0	195	195
PCB189	0	1272	1272	0	183	183
total^d^	507	2347	2536	192	2057	2196

a Abbreviation: NA, not applicable [due to no chlorinated compound identified as related compound for the corresponding targeted dioxin(-like) compound].

b The number of features associated with targeted or related dioxin(-like) compound under FDR 20%, adjusted by age, BMI, and factory.

c Features significantly associated with at least one related compound, which were significantly correlated with the specific targeted dioxin(-like) compound.

d Total number of unique features.

### 3.3 Annotations of Metabolic Features in Response to Dioxin(-like) Exposures

Among the metabolic features showing significant associations with dioxin(-like) exposures, level 1 annotations included 21 amino acids, 9 fatty acids, 4 cofactors, d-glucose, cholesterol, uric acid, and xanthine (Table S7). Further annotations at level 4 confidence included 7 androstane steroids and 8 metabolites from glycerolipids and glycerophospholipids.

In terms of metabolic pathway annotations, there were 33 pathways enriched from the metabolic features linked with PCDD(-related) compounds, 38 pathways with PCDF(-related) compounds, and 27 pathways with PCB(-related) compounds (Table 3). Among these, 21 pathways showed shared enrichment across all three subclasses, including 7 lipid pathways (de novo fatty acid biosynthesis, fatty acid activation, fatty acid metabolism, linoleate metabolism, phytanic acid peroxisomal oxidation, omega-3 fatty acid metabolism, and phosphatidylinositol phosphate metabolism), 6 amino acid pathways (alanine and aspartate, arginine and proline, aspartate and asparagine, histidine, lysine, and urea cycle/amino group metabolism), 3 carbohydrate pathways (amino sugar, butanoate, and pentose and glucuronate interconversion), purine metabolism, vitamin B6 metabolism, glutathione metabolism, drug metabolism, and xenobiotics metabolism.

**Table 3:**
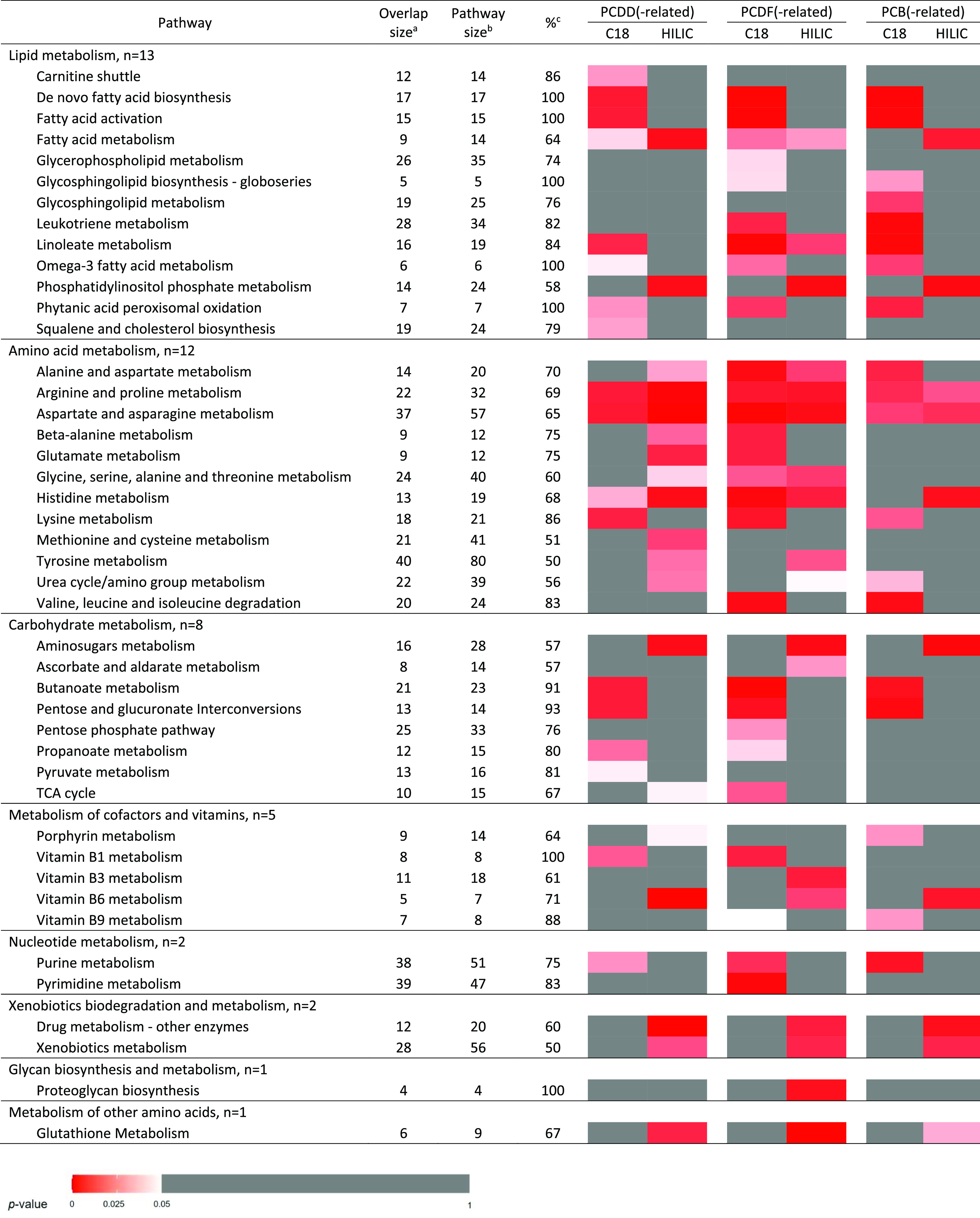
Enriched Biological Pathways Associated with Dioxin(-like) Exposures

a The average number of significant putative metabolites that were associated with dioxin(-like) exposures among each metabolic pathway.

b The average number of metabolites detected in each metabolic pathway.

c The percentage of overlap size to pathway size.

### 3.4 Integration of Metabolic Pathways with Immune Phenotypic End Points

The integration analysis involving metabolic pathways and immune end points was conducted only among factory A workers. Since the pathways largely overlapped for the three subclasses of dioxin(-like) exposures, integration analysis was first done involving pathways associated with all exposures and then moved on to analyses including pathways associated with PCDD(-related), PCDF(-related), and PCB(-related) compounds, separately.

**Figure 3:**
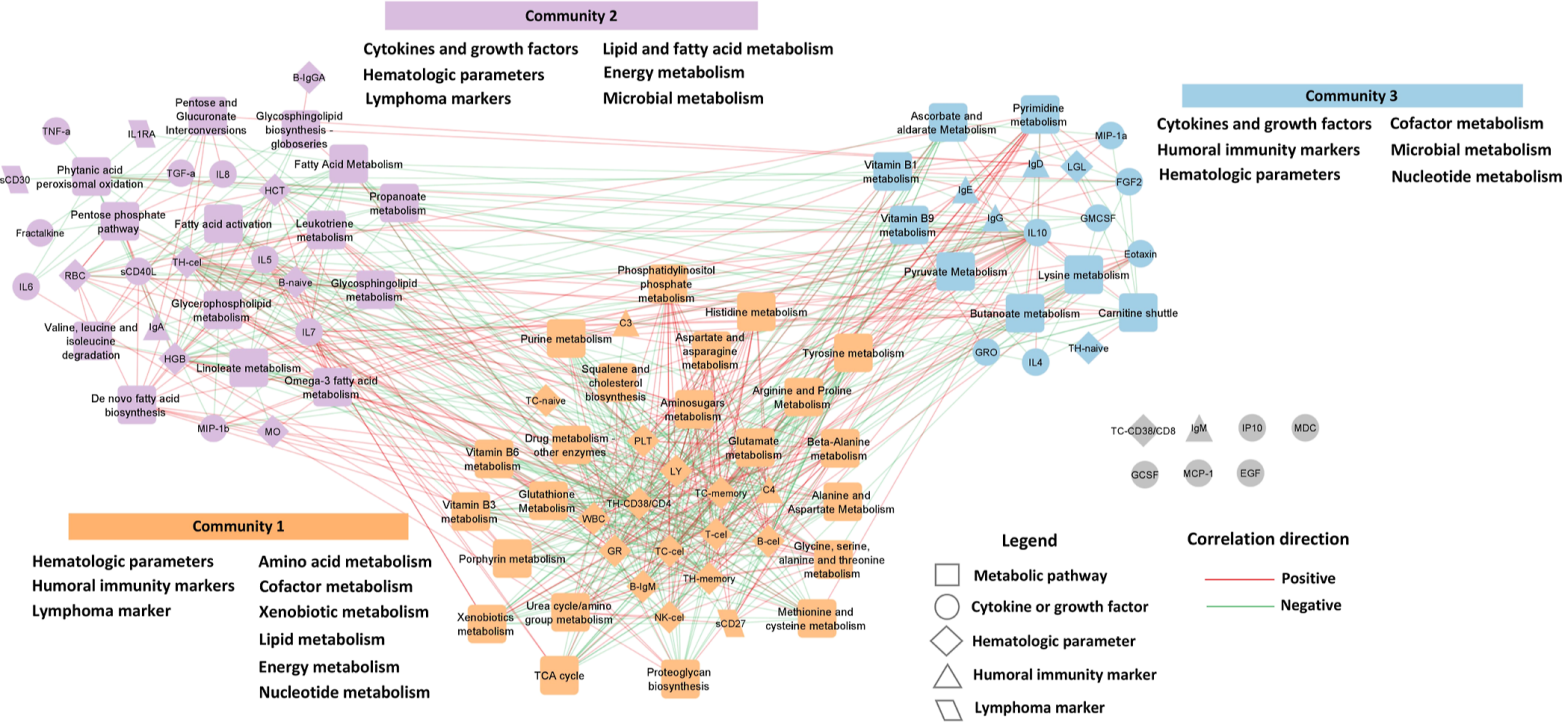
Network analysis of dioxin(-like)-related pathways and immune markers. Abbreviation: IL, interleukin; GMCSF, granulocyte-macrophage colony-stimulating factor; GCSF, granulocyte colony-stimulating factor; TNF-α, tumor necrosis factor alpha; EGF, epidermal growth factor; FGF2, fibroblast growth factor 2; GRO, melanoma growth stimulatory activity/growth-related oncogene; IP10, interferon gamma-induced protein 10; MCP-1, monocyte chemotactic protein-1; MDC, macrophage derived chemokine; MIP-1α, macrophage inflammatory protein-1 alpha; MIP-1β, macrophage inflammatory protein-1 beta; sCD40L, soluble CD40 ligand; TGF-α, transforming growth factor alpha; sCD30, soluble CD30; sCD27, soluble CD27; IL1RA, interleukin 1 receptor antagonist; RBC, red blood cells; HGB, hemoglobin; HCT, hematocrit; PLT, platelet counts; MO, monocytes; GR, granulocytes; LY, lymphocytes; B-cel, B cells; B-naïve, naïve B cells; B-IgM, IgM+ memory B cells; B-IgG, IgG/IgA+ memory B cells; T-cel, T cells; TH-cell, T helper cells; TH-CD38/CD4, CD38/CD4 cells; TH-naïve, naïve CD4 cells; TH-memory, memory CD4 cells; TC-cel, cytotoxic T cells; TC-CD38/CD8, CD38/CD8 cells; TC-naïve, naïve CD8 cells; TC-memory, memory CD8 cells; LGL, large granular lymphocytes; and NK-cel, natural killer cells.

In the network encompassing all pathways, every pathway (represented by PC1 scores) was associated with at least one immune marker (Figure 3). Community detection revealed the presence of three communities. Community 1 consisted of subsets of T and B lymphocytes, alongside complement factors (C3 and C4) and a lymphoma marker, soluble B-cell activation marker 27 (sCD27). Pathways associated with this community mainly included various amino acid pathways, the cofactor metabolism pathway (vitamin B3, B6, and porphyrin), xenobiotic metabolism pathways, and pathways of the purine and tricarboxylic acid (TCA) cycle. Community 2 predominantly included various cytokines and growth factors (mainly interleukins), hematologic parameters (red blood cells, hemoglobin, hematocrit, monocytes, B cells, and T helper cells), and lymphoma markers [soluble CD30 (sCD30) and interleukin 1 receptor antagonist (IL1RA)]. This community also exhibited associations with many lipid and fatty acid pathways, glucose metabolism pathways (pentose phosphate, pentose, and glucuronate interconversions), and the propanoate pathway. A smaller cluster, Community 3, included cytokines and growth factors, immunoglobins (IgD, IgE, and IgG), hematologic parameters [naïve CD4 cells, large granular lymphocytes (LGL)], clustered with pathways of cofactors (vitamins B1 and B9), pyruvate, and butanoate.

For networks specifically for each dioxin(-like) subclass, three communities were identified for both networks for PCDD(-related) and PCB(-related) compounds, and compositions were similar to those in the network for all exposures (Figure S8A,C). Whereas, in the case of PCDF(-related) compounds, the network yielded four communities (Figure S8B).

## 4 Discussion

In this study, we employed a pioneering approach that integrates chemical-wide and metabolome-wide analyses. The rationale for using chemical-wide analyses is that important biological insights might be missed by neglecting associated chemicals or related metabolites. This work was motivated by our previous observation in a MWAS on TCE conducted by Walker et al.^5^ In that study, it was observed that most exposure-related biological effects exhibited stronger associations with previously unidentified metabolic products of TCE rather than with TCE itself or recognized precursor metabolites. Similarly, in the present study, by including dioxin(-like)-related compounds, we have obtained a much richer insight into the associated biological responses. This finding challenges the prevailing paradigm of evaluating the toxic effects of chemicals solely by examining parent compounds and potentially recognized metabolites.^6^

It is important to note that applying this chemical-wide approach to different chemicals in other studies may not be straightforward. We acknowledge the uniqueness of the TCE and dioxin(-like) instances, wherein the identification of halogenated signals in untargeted HRMS analysis and prior knowledge of occupational exposure to parent compounds enabled us to identify related compounds and their metabolic products. However, considering the progress in HRMS data annotation capabilities through authentic standards and various in silico tools, the feasibility of this approach is likely to extend to other chemical-classes in the future. Our findings in this study underscore the potential benefits of this strategy, suggesting that it could lead to an enhanced assessment of toxicological effects.

The activation of the aryl hydrocarbon receptor (AhR) stands as a well-established mechanism of action for dioxin(-like) compounds.^38^ While numerous studies have investigated AhR-linked gene and protein expression,^38^ the underlying metabolic mechanism within the human body has received limited exploration. In an earlier study, we used nuclear magnetic resonance spectroscopy for metabolomic analysis on the same study population.^39^ Like our current investigation, this earlier analysis yielded few signals associated with the targeted TCDD (no. of features = 27, *p* < 0.05; none survived after multiple testing corrections). Jeanneret et al. identified 24 metabolites as putative biomarkers of dioxin exposures among TCDD-exposed workers.^40,41^ Liang et al., comparing HRMS for a high and low TCDD-exposed group, identified 20 metabolites strongly correlated to the summed toxicological equivalent quantity scores of 17 congeners of 2,3,7,8-substituted dioxins.^42^ In our enriched analyses, including dioxin(-like)-related compounds, we identified over 7,000 HRMS signals, underscoring the potency of integrating chemical-wide and metabolome-wide analyses.

Oxidative stress has been identified as a key mechanism underlying the toxicity of dioxin(-like) compounds,^38^ with biomolecules such as DNA, proteins, and lipids becoming targets of free radical attacks.^43^ In our study, a pathway enrichment analysis strongly suggests effects related to oxidative stress. The results point toward disruption in nucleotide metabolism, highlighted by the observation that PCDD(-related) and PCB(-related) compounds were linked to the purine pathway, while PCDF(-related) compounds were associated with both the purine and pyrimidine pathways. Seven amino acids susceptible to oxidative damage, annotated at level 1 (methionine, cysteine, lysine, proline, threonine, histidine, and tyrosine), were found to be associated with at least one of the three subclasses of dioxin(-like) exposures, and these associations were confirmed through enrichment analysis. Perturbations in lipids, encompassing membrane lipids (pathways of phospholipids, glycolipids, and cholesterol) and long-chain polyunsaturated fatty acids (omega-3 fatty acid pathway and linoleic acid with level 1 annotation), provide additional support for oxidation-induced lipid peroxidation. Conversely, reductions in the antioxidants, specifically glutathione and ascorbic acid pathways, further support heightened oxidative stress.

Carcinogenesis stands as the most severe outcome of dioxin(-like) toxicity, with TCDD, PCB126, and 23478F being classified as human carcinogens.^44^ In our study, pathways involving pyruvate (a glycolysis product), pentose phosphate, and the TCA cycle exhibited associations with both PCDD(-related) and PCDF(-related) compounds. Fatty acid pathways, encompassing biosynthesis, transport, activation, and degradation, demonstrated relations to all three subclasses of dioxin(-like) exposures. These aberrations in bioenergetic synthesis and fatty acid metabolism are consistent with microenvironmental shifts in human malignancies.

Our study also presents novel evidence of dysregulations within several metabolic pathways associated with dioxin(-like) exposures. Particularly noteworthy are microbiome-related pathways involving butanoate and propanoate and cofactor metabolisms, including porphyrin and B vitamins. Animal studies have indicated that exposure to TCDD and PCB126 can induce alterations in gut microbial composition.^45,46^ Additionally, PCB126 has been linked to elevated gut inflammation,^46^ while TCDD administration exhibited a mitigating effect on gut inflammation.^47^ The alteration of cofactors in response to dioxin-like compounds remains unexplored in experimental studies. The interplay between environmental dioxin(-like) compounds, the microbiome, and cofactors calls for further investigation.

Adverse immunological effects have been extensively documented in experimental studies.^48^ However, human data remains inconclusive. To investigate the immune toxicity of dioxin(-like) exposures, we performed integrative network analysis, connecting perturbed metabolic pathways and phenotypic measures of immune responses from samples of highly TCDD-exposed workers. In the network incorporating all dioxin(-like)-related pathways, distinct subsets of lymphocytes were grouped in the same community and linked to antioxidant pathways involving methionine, cysteine, and glutathione. Previous studies have shown that TCDD can suppress the differentiation of CD4^+^ T cells into effector cells^49^ and potently inhibit IgM production.^50^ Our findings suggest that oxidative stress could potentially underlie immune toxicity. As expected, relevant measures of cytokines and growth factors clustered together and exhibited enrichment with two inflammation-related pathways, linoleate, and leukotriene. Additionally, pathways related to fatty acid metabolism, bioenergy production, and gut microbiome were clustered with B-cell activation markers shown to be predictive of lymphoma risk. This highlights the potential role of immune responses in dioxin-like-induced carcinogenesis and microbiome dysbiosis.

We acknowledge several limitations in our study. First, this study adopts a cross-sectional design. Consequently, we cannot infer the temporal sequence of exposure and the health outcomes. Nonetheless, due to the protracted elimination of dioxin(-like) compounds, the measured levels effectively represent historical exposures. Second, the workers in factory A were, on average, 10 years older than the workers in factory B, and it is possible that other unmeasured factors differed between factories. However, in subgroup analyses by factory, the associations for dioxin(-like) compounds and metabolic features remained highly consistent with those in the main analysis, which included workers from both factories (Figure S9). Therefore, we conclude that characteristics specific to each factory did not substantially impact the effects of dioxin(-like) exposures in our presented analyses. Third, over the course of an extended 35 year follow-up period in the Dutch herbicide cohort, 27% of participants had died (567 out of 2,106 workers), and 5% were lost to follow-up (109 out of 2,106).^10^ This attrition may introduce the “healthy worker effect”, which may result in underestimating the adverse effects attributed to dioxin(-like) exposures. Fourth, precise annotations and absolute quantification of dioxin-like-related compounds continue to pose challenges. This limitation also impedes ascertaining these toxic chemicals’ origin, whether they originate from the environment or from endogenous metabolic modification. Therefore, future studies are necessary. Lastly, the study assessed targeted and untargeted dioxin-like exposures in two separate laboratories, without accounting for potential measurement variations between different analytical pipelines. Additionally, the untargeted compounds were not normalized for the lipid content. In a sensitivity analysis of MWAS on untargeted compounds, we further adjusted for total lipid levels measured at the time of the targeted measurement. The resulting altered features were similar to those generated in this study (data not shown). This suggests that the lipid content did not considerably bias our findings.

We employed a pioneering approach that integrates chemical-wide and metabolome-wide analyses. This innovative approach substantially broadens the ability to evaluate the biological effects of chemical exposures, encompassing not only the traditionally recognized dioxin(-like) compounds but also all relevant compounds representing co-exposures and exposure metabolites. The results from the MWAS align with the existing understanding of dioxin(-like) toxicities, highlighting perturbations in metabolic pathways linked to amino acids, lipid and fatty acids, carbohydrates, and nucleotides. Importantly, our study offers new perspectives regarding the mechanisms of action of dioxin(-like) compounds, such as altered activities of the gut microbiome.

## Supporting Information Available

Identification of dioxin(-like)-related compounds; descriptive statistics of targeted dioxin(-like) compounds; number of significant correlations between targeted dioxin(-like) compounds and chlorinated compounds under different cutoffs of *p*-value; list of immune phenotypic markers; number of dioxin(-like)-related compounds in relation to each targeted compound; targeted and related dioxin(-like) compounds in each community from network analysis; dioxin(-like)-related compounds with abundant associated metabolic features (*n* > 300); annotation of metabolic features associated with dioxin(-like) exposures; correlations of the same pathways across different dioxin(-like) subclasses; correlation heatmap of targeted dioxin(-like) compounds and all detected chlorinated compounds; correlations between targeted dioxin(-like) compounds and their related compounds; correlation matrix of targeted dioxin(-like) compounds; Venn diagram of PCDD-related, PCDF-related, and PCB-related compounds; Manhattan plots of MWAS of maximum TCDD levels; Manhattan plots of MWAS of certain dioxin(-like)-related compounds; Venn diagram of metabolic features associated with PCDD(-related), PCDF(-related), and PCB(-related) compounds; network analysis of dioxin(-like)-related pathways with immune markers, separately by PCDD(-related), PCDF(-related), and PCB(-related) compounds; and comparison of main and subgroup analyses by factory (PDF)
