# Supplementary material for: Dioxin(-like)-Related Biological Effects through Integrated Chemical-wide and Metabolome-wide Analyses
Source: Environ Sci Technol. 2023 Dec 27;58(1):258–68. doi: 10.1021/acs.est.3c07588 (PMC10785760; doi:10.1021/acs.est.3c07588)
Supplement: Supplementary file 1 — es3c07588_si_001.pdf [file es3c07588_si_001.pdf]

Supporting Information

Dioxin(-like) related biological effects through integrated chemical-wide and metabolome-wide analyses

Authors:

Yujia Zhao[1], Jeroen Meijer[1,2], Douglas I. Walker[3], Juni Kim[3], Lützen Portengen[1], Dean P. Jones[4], Fatemeh Saberi Hosnijeh[1], Jelle Vlaanderen[1], Roel Vermeulen[1,5]

Affiliations:

[1] Institute for Risk Assessment Sciences, Utrecht University, Utrecht 3584 CM, the Netherlands

[2] Department Environment & Health, Vrije Universiteit, Amsterdam 1081 HV, The Netherlands

[3] Gangarosa Department of Environmental Health, Rollins School of Public Health, Emory University, Atlanta, Georgia 30322, United States

[4] Division of Pulmonary, Allergy, Critical Care and Sleep Medicine, School of Medicine, Emory University, Atlanta, Georgia 30322, United States

[5] Julius Center for Health Sciences and Primary Care, University Medical Centre Utrecht, Utrecht 3584 CX, The Netherlands

Corresponding author:

Roel Vermeulen

Affiliation: Institute for Risk Assessment Sciences, Utrecht University, Utrecht 3584 CM, The Netherlands; Julius Center for Health Sciences and Primary Care, University Medical Centre Utrecht, Utrecht 3584 CX, The Netherlands

Postal address: Nieuw Gildestein, room 3.53, Yalelaan 2, 3584 CM, Utrecht, the Netherlands

e-mail address: R.C.H.Vermeulen@uu.nl

Number of Pages: 30

Number of Figures: 9

Number of Tables: 8

Supplemental content

Text S1. Identification of dioxin(-like) related compounds

Table S1. Descriptive statistics of targeted dioxin(-like) compounds

Table S2. The number of significant correlations between targeted dioxin(-like) compounds and chlorinated compounds under different cutoffs of p-value

Table S3. List of immune phenotypic markers

Table S4. The number of dioxin(-like) related compounds in relation to each targeted compound

Table S5. Targeted and related dioxin(-like) compounds in each community from network analysis

Table S6. Dioxin(-like) related compounds with abundant associated metabolic features (n>300)

Table S7. Annotation of metabolic features associated with dioxin(-like) exposures

Table S8. Correlations of the same pathways across different dioxin(-like) subclasses

Figure S1. Correlation heatmap of targeted dioxin(-like) compounds and all detected chlorinated compounds

Figure S2. Correlations between targeted dioxin(-like) compounds and their related compounds

Figure S3. Correlation matrix of targeted dioxin(-like) compounds

Figure S4. Venn diagram of PCDD-related, PCDF-related and PCB-related compounds

Figure S5. Manhattan plots of MWAS of maximum TCDD levels

Figure S6. Manhattan plots of MWAS of certain dioxin(-like) related compounds

Figure S7. Venn diagram of metabolic features associated with PCDD(-related), PCDF(-related) and PCB(-related) compounds

Figure S8. Network analysis of dioxin(-like) related pathways with immune markers, separately by PCDD(-related) (A), PCDF(related) (B), and PCB(-related) compounds (C)

Figure S9. Comparison of main analyses and subgroup analyses by factory

Text S1. Identification of dioxin(-like) related compounds

Approximately 60% of the pairwise Spearman correlations between the 499 chlorinated compounds detected by untargeted GC-HRMS and the 29 targeted dioxin(-like) compounds exhibited positive relationships (8,427 out of 14,471 correlations) (Figure S1). When considering the *p*-value cutoff as 0.05, the average proportion of negative correlations across all targeted dioxin(-like) compounds was 40% (Table S2). Notably, compounds displaying negative correlations to targeted compounds are unlikely to be co-exposures or metabolites of parental compounds. As such, the percentage of negatively correlated significant outcomes can be regarded as empirically derived from false discovery rates. For the *p*-value cutoff of 0.002, the average proportion of negative correlations was around 20%.

Applying the criteria of correlation exceeding 0 and *p*-value below 0.002, we identified 152 chlorinated compounds that exhibited correlations with 23 targeted dioxin(-like) compounds (Figure S2). These correlation coefficients ranged from 0.26 to 0.75, with a median of 0.33. Among these, 106 chlorinated compounds were correlated to more than one targeted dioxin(-like) compound, and 45 compounds were related to more than five targeted compounds.

Table S1. Descriptive statistics of targeted dioxin(-like) compounds

Compound	Factory A (n=76)[a]	Factory B (n=61)[a]	p-value[b]	Reference level[a,c]
PCDDs				
TCDD	4.35 (1.60-5.60)	0.30 (0.21-0.56)	<0.0001	<LOD
12378D	7.55 (6.50-9.10)	6.40 (5.30-7.00)	0.016	<LOD
123478D	4.50 (3.60-5.40)	1.90 (1.11-3.10)	<0.001	<LOD
123678D	30.0 (27.2-33.1)	20.7 (15.6-25.7)	<0.001	19.8 (17.8-21.6)
123789D	3.70 (2.70-4.60)	0.62 (0.42-1.06)	<0.0001	<LOD
1234678D	15.6 (12.5-18.1)	12.5 (9.6-14.8)	0.041	23.2 (21.1-25.6)
OCDD	216 (192-269)	171 (139-212)	0.036	<LOD
Dioxin-like PCDFs				
2378F	0.02 (0.01-0.03)	0.02 (0.01-0.04)	0.523	<LOD
12378F	0.14 (0.10-0.19)	0.14 (0.12-0.21)	0.342	<LOD
23478F	22.9 (20.5-27.5)	13.1 (11.6-15.4)	<0.0001	<LOD
123478F	4.75 (4.10-5.70)	4.50 (3.70-4.90)	0.091	<LOD
123678F	5.65 (5.00-6.20)	4.90 (4.20-5.70)	0.059	<LOD
123789F	0.39 (0.39-0.40)	0.39 (0.39-0.40)	0.936	<LOD
234678F	0.30 (0.14-0.55)	0.11 (0.07-0.17)	0.008	<LOD
1234678F	4.30 (3.70-5.20)	4.10 (3.20-5.90)	0.990	<LOD
1234789F	0.10 (0.07-0.15)	0.11 (0.07-0.16)	0.827	<LOD
OCDF	0.68 (0.38-1.80)	0.49 (0.31-0.72)	0.512	<LOD
Dioxin-like PCBs				
PCB77	23.7 (20.4-26.6)	23.6 (20.0-29.2)	0.940	NA
PCB81	4.95 (3.80-6.50)	6.80 (6.00-7.90)	<0.001	<LOD
PCB126	38.8 (35.0-46.2)	37.0 (29.4-42.3)	0.276	<LOD
PCB169	83.3 (78.2-90.1)	63.1 (54.9-74.0)	<0.0001	<LOD
PCB105	1.55 (1.20-1.80)	1.40 (1.10-1.90)	0.661	0.98 (0.85-1.08)
PCB114	1.00 (0.80-1.10)	0.80 (0.60-1.10)	0.379	NA
PCB118	11.0 (9.2-13.0)	10.0 (6.7-12.9)	0.557	4.71 (4.16-5.06)
PCB123	0.23 (0.20-0.30)	0.21 (0.15-0.27)	0.479	NA
PCB156	13.9 (12.6-15.9)	12.4 (10.0-14.6)	0.076	3.10 (2.80-3.80)
PCB157	2.50 (2.30-3.00)	2.30 (1.70-2.80)	0.177	0.75 (0.70-0.90)
PCB167	3.40 (2.90-3.90)	2.80 (2.00-3.80)	0.296	0.50 (0.44-0.60)
PCB189	2.30 (2.10-2.50)	1.90 (1.70-2.20)	0.008	<LOD
$TCDD_{max}$[d]	98.9 (19.2-341.6)	0.30 (0.21-0.56)	<0.0001	

Abbreviations: PCDD, pentachloro dibenzo-p-dioxin; TCDD, 2,3,7,8-tetrachloro dibenzo-p-dioxin; 12378D, 1,2,3,7,8-PCDD; 123478D, 1,2,3,4,7,8-PCDD; 123678D, 1,2,3,6,7,8-PCDD; 123789D, 1,2,3,7,8,9-PCDD; 1234678D, 1,2,3,4,6,7,8-PCDD; OCDD, octachloro dibenzo-p-dioxin; PCDF, pentachloro dibenzofuran; 2378F, 2,3,7,8- tetrachloro dibenzofuran; 12378F, 1,2,3,7,8- PCDF; 23478F, 2,3,4,7,8-PCDF; 123478F, 1,2,3,4,7,8-hexachloro dibenzofuran (HCDF); 123678F, 1,2,3,6,7,8-HCDF; 123789F, 1,2,3,7,8,9-HCDF; 234678F, 2,3,4,6,7,8-HCDF; 1234678F, 1,2,3,4,6,7,8-heptachloro dibenzofuran (HpCDF); 1234789F, 1,2,3,4,5,8,9-HpCDF; OCDF, octachloro dibenzofuran; PCB, polychlorinated biphenyls; PCB77, 3,3',4,4'-tetrachlorobiphynyl (tetraCB); PCB81, 3,4,4',5-tetraCB; PCB126, 3,3',4,4',5-pentachlorobiphenyl (pentaCB); PCB169, 3,3',4,4',5,5'-hexachlorobiphenyl (hexaCB); PCB105, 2,3,3',4,4'-pentaCB; PCB114, 2,3,4,4',5-pentaCB; PCB118, 2,3',4,4',5-pentaCB;

PCB123, 2’,3,4,4’,5-pentraCB; PCB156, 2,3,3’,4,4’,5-hexaCB; PCB157, 2,3,3’,4,4’,5’-hexaCB; PCB167, 2,3’,4,4’,5,5’-hexaCB; PCB189, 2,3,3’,4,4’,5,5’-heptaCB; LOD, limit of detection; NA, not available.

[a]Parts per trillion, lipid adjusted; data are presented as median (95% confidence interval)

[b]p-value from Mann-Whitney-Wilcoxon test, concentrations between factory A vs. factory B

[c]Concentrations of dioxin(-like) compounds from US general males in 2003-2004, reported by National Health and Nutrition Examination Survey.

[d]Estimated maximum levels of TCDD

Table S2. The number of significant correlations between targeted dioxin(-like) compounds and chlorinated compounds under different cutoffs of *p*-value

Targeted compound	*p*-value<0.05		*p*-value<0.01		*p*-value<0.002		*p*-value<0.001		*p*-value<0.00005	
	No.[a]	%[b]	No.[a]	%[b]	No.[a]	%[b]	No.[a]	%[b]	No.[a]	%[b]
PCDDs										
TCDD	198/31	16	121/25	21	74/21	28	61/17	28	18/9	50
12378D	170/31	18	97/14	14	52/12	23	45/11	24	21/4	19
123478D	171/31	18	94/17	18	51/13	25	36/10	28	11/2	18
123678D	192/27	14	124/20	16	73/11	15	59/8	14	23/4	17
123789D	163/20	12	73/6	8	35/5	14	26/4	15	3/1	33
1234678D	111/17	15	36/5	14	6/1	17	4/0	0	1/0	0
OCDD	134/17	13	50/8	16	18/1	6	10/1	0	1/0	0
Dioxin-like PCDFs										
2378F	24/18	75	3/2	67	none	NA	none	NA	none	NA
12378F	48/31	65	6/2	33	1/0	0	none	NA	none	NA
23478F	263/37	14	202/23	11	149/19	13	137/19	14	85/13	15
123478F	139/33	24	69/15	22	28/3	11	20/2	10	7/0	0
123678F	115/57	50	52/23	44	34/15	44	27/11	41	11/2	18
123789F	8/6	75	2/2	100	none	NA	none	NA	none	NA
234678F	130/13	10	63/3	5	28/1	4	17/1	6	none	NA
1234678F	43/18	42	8/3	38	2/1	50	1/0	0	none	NA
1234789F	37/31	84	6/6	100	1/1	100	1/1	100	none	NA
OCDF	1/1	100	none	NA	none	NA	none	NA	none	NA
Dioxin-like PCBs										
PCB77	6/6	100	none	NA	none	NA	none	NA	none	NA
PCB81	136/128	94	56/53	95	16/16	100	10/10	100	none	NA
PCB126	84/52	62	28/11	39	15/3	20	13/2	15	4/0	0
PCB169	206/28	14	138/25	18	70/18	26	51/13	25	19/1	5
PCB105	64/33	52	28/8	29	23/3	13	22/2	9	14/0	0

PCB114	105/18	17	48/6	12	29/0	0	25/0	0	19/0	0
PCB118	76/40	53	39/11	28	23/3	13	22/2	9	16/0	0
PCB123	30/5	17	18/1	6	8/0	0	8/0	0	3/0	0
PCB156	119/20	17	51/9	18	29/5	17	24/1	4	21/0	0
PCB157	90/16	18	41/8	20	27/4	15	23/1	4	20/0	0
PCB167	113/54	48	44/16	36	26/2	8	25/1	4	20/0	0
PCB189	103/19	18	39/9	23	25/1	4	23/0	0	19/0	0
Average %[c]	40		32		22		19		9	

Abbreviation: NA, not applicable.

p-values were calculated from tests of pairwise Spearman correlations between known dioxin(-like) compounds and chlorinated compounds.

[a]No. of all significant associations under the p-value cutoff / No. of negative and significant associations under the p-value cutoff.

[b]The percentage of negative associations among all significant associations.

[c]The average percentage of negative associations among all known dioxin(-like) compounds.

Table S3. List of immune phenotypic markers

Full name	Acronym
Cytokines and growth factors	
Interleukin 4	IL4
Interleukin 5	IL5
Interleukin 6	IL6
Interleukin 7	IL7
Interleukin 8	IL8
Interleukin 10	IL10
Granulocyte-macrophage colony-stimulating factor	GMCSF
Granulocyte colony-stimulating factor	GCSF
Tumor necrosis factor alpha	TNF-a
Epidermal growth factor	EGF
Eotaxin	Eotaxin
Fibroblast growth factor 2	FGF2
Fractalkine	Fractalkine
Melanoma growth stimulatory activity/growth-related oncogene	GRO
Interferon gamma-induced protein 10	IP10
Monocyte chemotactic protein-1	MCP-1
Macrophage derived chemokine	MDC
Macrophage inflammatory protein-1 alpha	MIP-1a
Macrophage inflammatory protein-1 beta	MIP-1b
Transforming growth factor alpha	TGF-a
Soluble CD40 ligand	sCD40L
Hematologic parameters	
Red blood cells	RBC
Hemoglobin	HGB
Hematocrit	HCT
Platelet count	PLT
White blood cells	WBC
Monocytes	MO
Granulocytes	GR
Lymphocytes	LY
B cells	B-cel
Naïve B cells	B-naive
IgM+ memory B cells	B-IgM
IgG/IgA+ memory B cells	B-IgGA
T cells	T-cel
T helper cells	TH-cel
CD38/CD4 cells	TH-CD38/CD4
Naïve CD4 cells	TH-naive
Memory CD4 cells	TH-memory
Cytotoxic T cells	TC-cel
CD38/CD8 T cells	TC-CD38/CD8
Naïve CD8 cells	TC-naive
Memory CD8 cells	TC-memory
Large granular lymphocytes	LGL

Natural killer cells	NK-cel
Humoral immunity markers	
Immunoglobulin A	IgA
Immunoglobulin D	IgD
Immunoglobulin E	IgE
Immunoglobulin G	IgG
Immunoglobulin M	IgM
Complement factor 3	C3
Complement factor 4	C4
Lymphoma markers	
Soluble CD30	sCD30
Soluble CD27	sCD27
Interleukin 1 receptor antagonist	IL1RA

Table S4. The number of dioxin(-like) related compounds in relation to each targeted compound

Targeted compound	n. of chlorinated related compounds[a]
PCDDs	
TCDD	53
12378D	40
123478D	38
123678D	62
123789D	30
1234678D	5
OCDD	17
Total	109
Dioxin-like PCDFs	
2378F	0
12378F	1
23478F	130
123478F	25
123678F	19
123789F	0
234678F	27
1234678F	1
1234789F	0
OCDF	0
Total	136
Dioxin-like PCBs	
PCB77	0
PCB81	0
PCB126	12
PCB169	52
PCB105	20
PCB114	29
PCB118	20
PCB123	8
PCB156	24
PCB157	23
PCB167	24
PCB189	24
Total	58

[a]Chlorinated compounds were considered in relation to targeted dioxin(-like) compounds when they exhibited a correlation coefficient exceeding 0 and *p*-value below 0.002 with at least one targeted compound.

Table S5. Targeted and related dioxin(-like) compounds in each community from network analysis

Targeted compounds	Related compounds
Community 1	
1234678D<br>123478F, 123678F<br>PCB126, PCB105, PCB114, PCB118, PCB123, PCB156, PCB157, PCB167, PCB189	C00014, C00026, C00036, C00038, C00047, C00050, C00068, C00149, C00150, C00206, C00268, C00269, C00508, C00586, C02267, C03500, C03641, C00031, C00428, C00430, C01742, C02696, C00077, C00094, C00459, C01193
Community 2	
12378D, 123478D, 123678D, 123789D, OCDD	C00028, C00239, C00320, C00461, C00764, C03231, C04165, C05116, C05225, C00035, C00189, C00272, C00294, C00391, C00535, C00555, C00582, C00691, C00737, C01785, C01911, C00922, C01192, C03354, C05219, C00071, C00568, C00609, C00630, C00883, C01058, C01362, C02246, C03335, C03698, C06046, C01023, C01070, C01761, C00160, C00897, C02490, C05131
Community 3	
TCDD<br>23478F, 234678F	C00087, C00295, C00324, C00556, C00660, C00712, C00810, C00915, C00926, C01101, C01128, C01215, C02000, C02113, C02226, C02624, C02642, C02652, C04306, C04781, C06136, C06162, C00052, C05187, C00018, C00039, C00241, C00307, C05104, C00644, C00738, C01659, C02110, C02614, C05649, C00033, C00044, C00045, C00064, C00109, C00113, C00134, C00139, C00161, C00221, C00466, C00570, C00649, C00795, C01120, C01141, C01436, C01480, C01685, C01812, C02269, C02655, C02671, C02871, C03365, C03428, C03951, C04160, C04163, C04182, C04244, C05499, C05657, C06286, C02945
Community 4	
PCB169	C00115, C01633, C02016, C03662, C06171, C00890, C02084, C00856, C00928, C00488, C00805
Community 5	
12378F	C04604
Community 6	
1234678F	C00066

Table S6. Dioxin(-like) related compounds with abundant associated metabolic features (n>300)

Related compound	Community[a]	Correlated known compounds[b]	No. of features[c]
		C18-negative	
C00295	3	TCDD	1179
C05187	3	123478D, 23478F, 123478F	952
C00459	1	123478F, PCB167	732
C02671	3	23478F, 234678F	679
C00430	1	12378D, 123678D, 23478F, 123678F, PCB169, PCB105, PCB114, PCB118 PCB156, PCB157, PCB167, PCB189	585
C00047	1	TCDD, 12378D, 123478D, 123678D, 23478F, PCB169, PCB105, PCB114, PCB118, PCB156, PCB157, PCB167, PCB189	567
C00044	3	23478F	565
C00764	2	TCDD, 12378D, 123478D, 123678D, 23478F, 234678F, PCB169	556
C00094	1	123478D, 123678D, 23478F, PCB169, PCB105, PCB114, PCB118, PCB156, PCB157, PCB167, PCB189	518
C02113	3	TCDD, 23478F	512
C01480	3	23478F	493
C00660	3	TCDD, 123678D, 23478F, 234678F, PCB169	458
C04182	3	23478F	431
C02945	3	234678F	411
C00795	3	23478F, 234678F	391
C00915	3	TCDD, 23478F	363
C00052	3	12378D, 123678D, 23478F, 234678F	352
C00139	3	23478F, 123478F	343
C00582	2	12378D, 23478F, 234678F	338
C00269	1	TCDD, 12378D, 123478D, 123678D, 23478F, 123478F, 123678F, PCB126, PCB169, PCB105, PCB114, PCB118, PCB156, PCB157, PCB167, PCB189	327
C00691	2	12378D, 123678D, OCDD, 23478F, 123478F, 234678F	310
C02016	4	TCDD, 23478F, PCB169	309
		HILIC-positive	
C00922	2	123478D	1464
C02084	4	123789D, 23478F, PCB169	1186
C00221	3	23478F	1158
C00035	2	12378D, 123478D, 123678D, 23478F, 123478F, PCB169, PCB114	565
C00066	6	1234678F	302

[a]Community from community detection of the network between known and related dioxin(-like) related compounds (Supplemental table 5).
[b]Under the criteria of Spearman correlation coefficient exceeding 0 and *p*-value below 0.002.
[c]The number of significantly associated features under FDR 20%, adjusted by age, BMI and factory.

Table S7. Annotation of metabolic features associated with dioxin(-like) exposures

Class	HMDB ID	Name	m/z	Retention time	Confidence level	Mode	Adduct	PCDD (-related)[a]	PCDF (-related)[a]	PCB (-related)[a]
amino acid	HMDB00001	1-methylhistidine	170.0924	82.3	1	HILIC	M+H	1	2	1
amino acid	HMDB00043	betaine	116.0717	240.4	1	C18	M-H	2	2	1
amino acid	HMDB00064	creatine	130.0605	151.2	1	C18	M-H	6	9	2
amino acid	HMDB00112	aminobutyrate	104.0706	61.8	1	HILIC	M+H	1	0	0
amino acid	HMDB00123	glycine	120.0032	67.8	1	HILIC	M+2Na-H	0	0	1
amino acid	HMDB00148	glutamic acid	151.0627	25	1	C18	M-H	1	5	1
			153.0772	70.4	1	HILIC	M+H	2	3	1
amino acid	HMDB00158	tyrosine	181.0638	30.2	1	C18	M-H	1	1	0
			183.0783	57.8	1	HILIC	M+H	0	1	0
			183.0804	86.3	1	HILIC	M+H	17	20	7
			183.0804	29.7	1	HILIC	M+H	2	2	0
			182.0812	53.9	1	HILIC	M+H	1	2	1
amino acid	HMDB00162	proline	114.056	289.6	1	C18	M-H	1	0	0
			116.0706	75	1	HILIC	M+H	1	0	0
amino acid	HMDB00167	threonine	120.0655	25.7	1	HILIC	M+H	0	1	0
			120.0655	68	1	HILIC	M+H	2	2	1
amino acid	HMDB00168	asparagine	131.0445	292.4	1	C18	M-H	11	16	4
			131.0445	182.8	1	C18	M-H	7	11	3
			133.0603	106.7	1	HILIC	M+H	1	1	1
amino acid	HMDB00177	histidine	154.0623	24.4	1	C18	M-H	0	1	0
			156.0768	82.2	1	HILIC	M+H	2	2	1
amino acid	HMDB00182	lysine	145.0983	32.4	1	C18	M-H	1	2	0
			147.1128	87.1	1	HILIC	M+H	3	3	2
amino acid	HMDB00192	cystine	241.0232	197.4	1	C18	M-H	2	2	1
			241.0233	34.2	1	C18	M-H	2	5	2
			243.0378	185.9	1	HILIC	M+H	0	1	0

amino acid	HMDB00214	ornithine	133.0971	91.2	1	HILIC	M+H	2	2	1
amino acid	HMDB00562	creatinine	112.0516	285.2	1	C18	M-H	9	14	4
			114.0662	38.7	1	HILIC	M+H	1	2	1
amino acid	HMDB00574	cysteine	122.027	114.1	1	HILIC	M+H	0	1	0
amino acid	HMDB00641	glutamine	145.0619	23.2	1	C18	M-H	0	0	1
			154.0862	92.3	1	HILIC	M+H	0	1	0
			154.0863	225.5	1	HILIC	M+H	2	8	1
			154.0863	29.1	1	HILIC	M+H	20	21	4
amino acid	HMDB00696	methionine	155.0374	187.3	1	C18	M-H	1	1	0
amino acid	HMDB00883	valine	116.0717	240.4	1	C18	M-H	2	2	1
amino acid	HMDB00904	citrulline	174.0885	22.9	1	C18	M-H	1	1	1
			176.103	80.6	1	HILIC	M+H	2	2	1
amino acid	HMDB00929	tryptophan	203.0827	27	1	C18	M-H	0	1	1
amino acid	HMDB06029	N-acetylglutamine	247.0934	28.1	2	C18	M+Hac-H	0	1	0
			233.0779	26.8	2	C18	M-H+HCOOH	0	1	0
			187.0724	27.8	2	C18	M-H	1	2	1
androstane steroid	HMDB00369	3b17b-dihydroxyetiocholane	351.2541	236	2	C18	M+Hac-H	8	11	6
			291.2328	233.1	2	C18	M-H	8	10	6
androstane steroid	HMDB00383	3a17a-dihydroxy-5b-androstane	351.2541	236	2	C18	M+Hac-H	8	11	6
			291.2328	233.1	2	C18	M-H	8	10	6
androstane steroid	HMDB00412	3b17a-dihydroxy-5a-androstane	351.2541	236	2	C18	M+Hac-H	8	11	6
			291.2328	233.1	2	C18	M-H	8	10	6
androstane steroid	HMDB00458	5a-androstane-3a17a-diol	351.2541	236	2	C18	M+Hac-H	8	11	6
			291.2328	233.1	2	C18	M-H	8	10	6
androstane steroid	HMDB00493	5a-androstane-3b17b-diol	351.2541	236	2	C18	M+Hac-H	8	11	6
			291.2328	233.1	2	C18	M-H	8	10	6
androstane steroid	HMDB00551	etiocholanediol	351.2541	236	2	C18	M+Hac-H	8	11	6
			291.2328	233.1	2	C18	M-H	8	10	6
androstane steroid	HMDB00554	dihydroandrosterone	351.2541	236	2	C18	M+Hac-H	8	11	6

			291.2328	233.1	2	C18	M-H	8	10	6
carbohydrate	HMDB00122	D-glucose	209.0728	57.7	1	HILIC	M+Na	0	1	0
cholestane steroid	HMDB00067	cholesterol	369.3511	26.2	1	HILIC	M+H-H2O	4	5	3
cofactor	HMDB00244	riboflavin	377.1454	43.7	1	HILIC	M+H	1	2	0
cofactor	HMDB01431	pyridoxamine	169.0948	84.8	1	HILIC	M+H	1	1	0
cofactor	HMDB01488	niacin	122.0247	289.1	1	C18	M-H	9	11	6
			124.0394	170.1	1	HILIC	M+H	2	1	1
			124.0394	12.4	1	HILIC	M+H	0	1	0
			124.0411	100.6	1	HILIC	M+H	2	1	1
cofactor	HMDB01545	pyridoxal	166.0511	109.4	1	C18	M-H	1	0	0
dipeptide	HMDB11173	glycylhydroxyproline	247.0934	28.1	2	C18	M+Hac-H	0	1	0
			233.0779	26.8	2	C18	M-H+HCOOH	0	1	0
			187.0724	27.8	2	C18	M-H	1	2	1
fatty acid	HMDB00201	Acetylcarnitine	204.1231	49.5	1	HILIC	M+H	2	1	1
fatty acid	HMDB00220	Palmitic acid	255.2328	251.8	1	C18	M-H	9	14	8
			257.2476	245.8	1	HILIC	M+H	1	1	0
			257.2477	19.5	1	HILIC	M+H	7	16	3
fatty acid	HMDB00222	Palmitoylcarnitine	400.3499	92.7	1	HILIC	M+H	2	2	0
			400.3499	254.2	1	HILIC	M+H	3	5	3
fatty acid	HMDB00511	Decanoic acid	171.139	246.9	1	C18	M-H	3	3	4
			171.1392	118.7	1	C18	M-H	6	5	2
fatty acid	HMDB00638	Lauric acid	199.1704	270	1	C18	M-H	2	1	1
fatty acid	HMDB00673	Linoleic acid	279.2327	238.7	1	C18	M-H	8	13	6
fatty acid	HMDB00827	Stearic acid	283.264	276.9	1	C18	M-H	8	12	6
			285.2788	25.8	1	HILIC	M+H	4	6	2
fatty acid	HMDB01388	Alpha-Linolenic acid	277.2172	224.1	1	C18	M-H	8	11	6
fatty acid	HMDB02212	Arachidic acid	311.2956	290.3	1	C18	M-H	7	7	6
glycerolipid	HMDB11131	MG(18:0/0:0/0:0)	376.3419	23.4	2	HILIC	M+NH4	3	4	2
			381.2973	28.2	2	HILIC	M+Na	3	4	2

			359.3152	25.8	2	HILIC	M+H	8	12	4
glycerolipid	HMDB11533	MG(0:0/16:0/0:0)	353.2662	22.2	2	HILIC	M+Na	1	2	2
			331.2839	26.1	2	HILIC	M+H	7	8	3
			376.3419	23.4	2	HILIC	M+NH4	3	4	2
			381.2973	28.2	2	HILIC	M+Na	3	4	2
			359.3152	25.8	2	HILIC	M+H	8	12	4
glycerolipid	HMDB11564	MG(16:0/0:0/0:0)	353.2662	22.2	2	HILIC	M+Na	1	2	2
			331.2839	26.1	2	HILIC	M+H	7	8	3
glycerophospholipid	HMDB00495	Androstanediol	351.2541	236	2	C18	M+Hac-H	8	11	6
			291.2328	233.1	2	C18	M-H	8	10	6
glycerophospholipid	HMDB11483	LysoPE(0:0/20:2)	564.3326	221.9	2	C18	M+Hac-H	0	1	1
			504.3092	223.5	2	C18	M-H	0	2	1
glycerophospholipid	HMDB11499	LysoPE(0:0/24:6)	612.3294	211.7	2	C18	M+Hac-H	0	0	1
			552.3092	211.6	2	C18	M-H	0	0	4
glycerophospholipid	HMDB11513	LysoPE(20:2/0:0)	564.3326	221.9	2	C18	M+Hac-H	0	1	1
			504.3092	223.5	2	C18	M-H	0	2	1
glycerophospholipid	HMDB11529	LysoPE(24:6/0:0)	612.3294	211.7	2	C18	M+Hac-H	0	0	1
			552.3092	211.6	2	C18	M-H	0	0	4
purine or its derivative	HMDB00289	Uric acid	167.0211	24.4	1	C18	M-H	7	10	4
purine or its derivative	HMDB00292	Xanthine	151.0262	25.1	1	C18	M-H	38	51	17

[a]The number of known and related dioxin(-like) compounds associated with the specific metabolite.

Table S8. Correlations of the same pathways across different dioxin(-like) subclasses

Pathway	PCDD vs. PCDF	PCDD vs. PCB	PCDF vs. PCB
De novo fatty acid biosynthesis	1.00	1.00	1.00
Aspartate and asparagine metabolism	0.99	0.99	0.99
Fatty acid activation	1.00	1.00	1.00
Arginine and Proline Metabolism	0.98	-0.98	-0.98
Pentose and Glucuronate Interconversions	0.98	0.97	0.98
Butanoate metabolism	0.93	-0.97	-0.88
Lysine metabolism	-0.97	0.95	-0.95
Linoleate metabolism	0.99	-1.00	-0.99
Phytanic acid peroxisomal oxidation	0.98	0.98	1.00
Purine metabolism	-1.00	0.99	-0.98
Histidine metabolism	0.99	-0.96	-0.96
Fatty Acid Metabolism	1.00	0.55	0.56
Omega-3 fatty acid metabolism	1.00	1.00	1.00
Drug metabolism - other enzymes	0.99	-0.99	-1.00
Vitamin B6 metabolism	1.00	1.00	1.00
Aminosugars metabolism	0.97	0.99	0.99
Phosphatidylinositol phosphate metabolism	0.99	-0.98	-0.98
Glutathione Metabolism	0.89	-0.97	-0.93
Xenobiotics metabolism	0.99	-0.99	-0.99
Urea cycle/amino group metabolism	0.98	-0.73	-0.74
Alanine and Aspartate Metabolism	-0.86	-0.62	0.82

PC1 scores for each pathway were calculated based on selected features in the corresponding pathway. For the same pathway, included features could be slightly different across the three dioxin(-like) subclasses. Correlation coefficients of the same pathway in different exposure subclasses were presented.

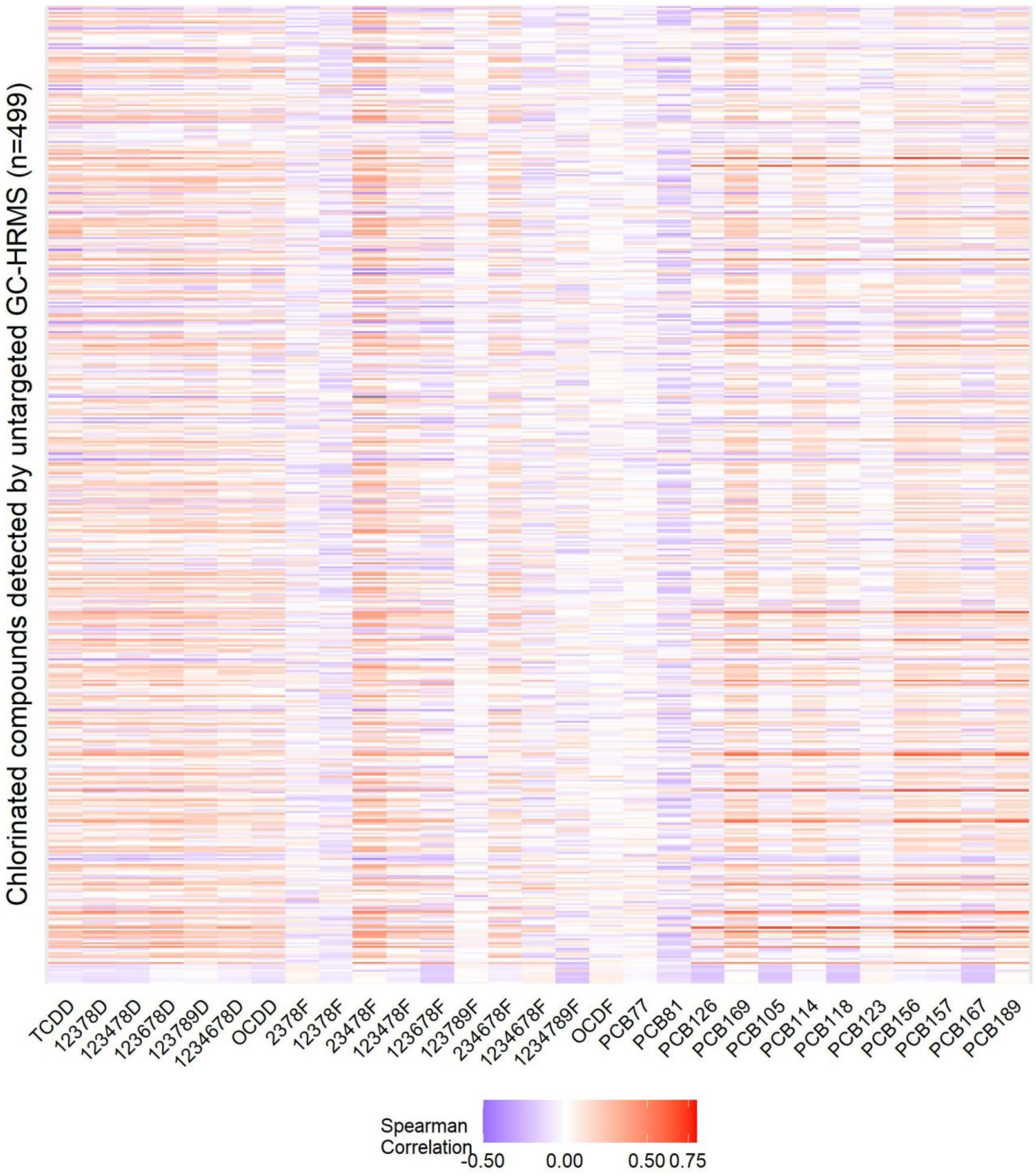

Figure S1. Correlation heatmap of targeted dioxin(-like) compounds and all detected chlorinated compounds

Tile color showed magnitude of pairwise Spearman correlation coefficients of the targeted dioxin(-like) compounds (n=29) and all chlorinated compounds detected by untargeted GC-HRMS (n=499).

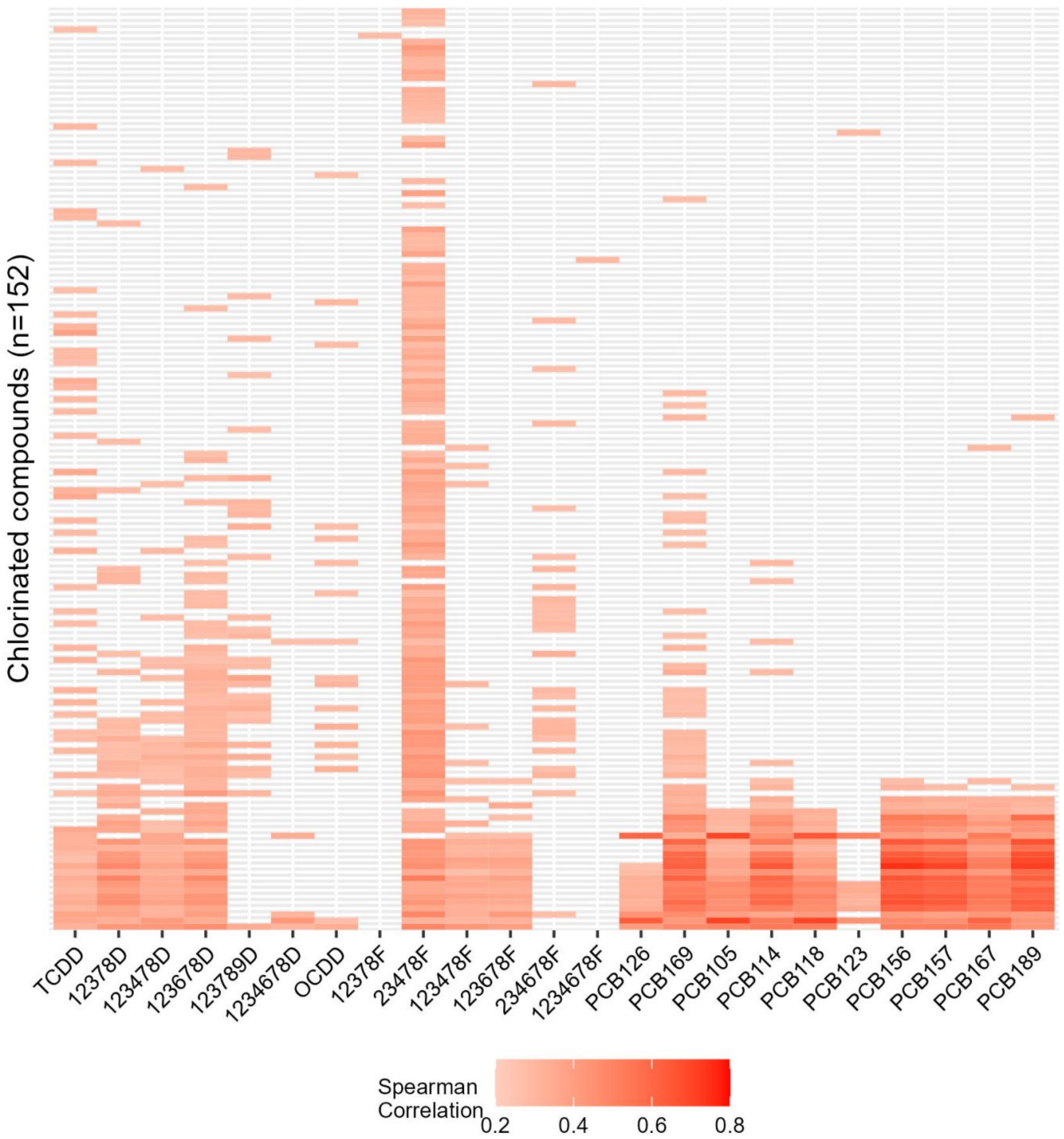

Figure S2. Correlations between targeted dioxin(-like) compounds and their related compounds

Chlorinated compounds (n=152) were identified as possible related dioxin(-like) compounds, under the criteria of pairwise correlation coefficient exceeding 0 and *p*-value below 0.002. Tile color showed magnitude of correlation coefficients; gray color indicated the correlation was either non-significant (*p*-value≥0.002) or in negative direction, or both.

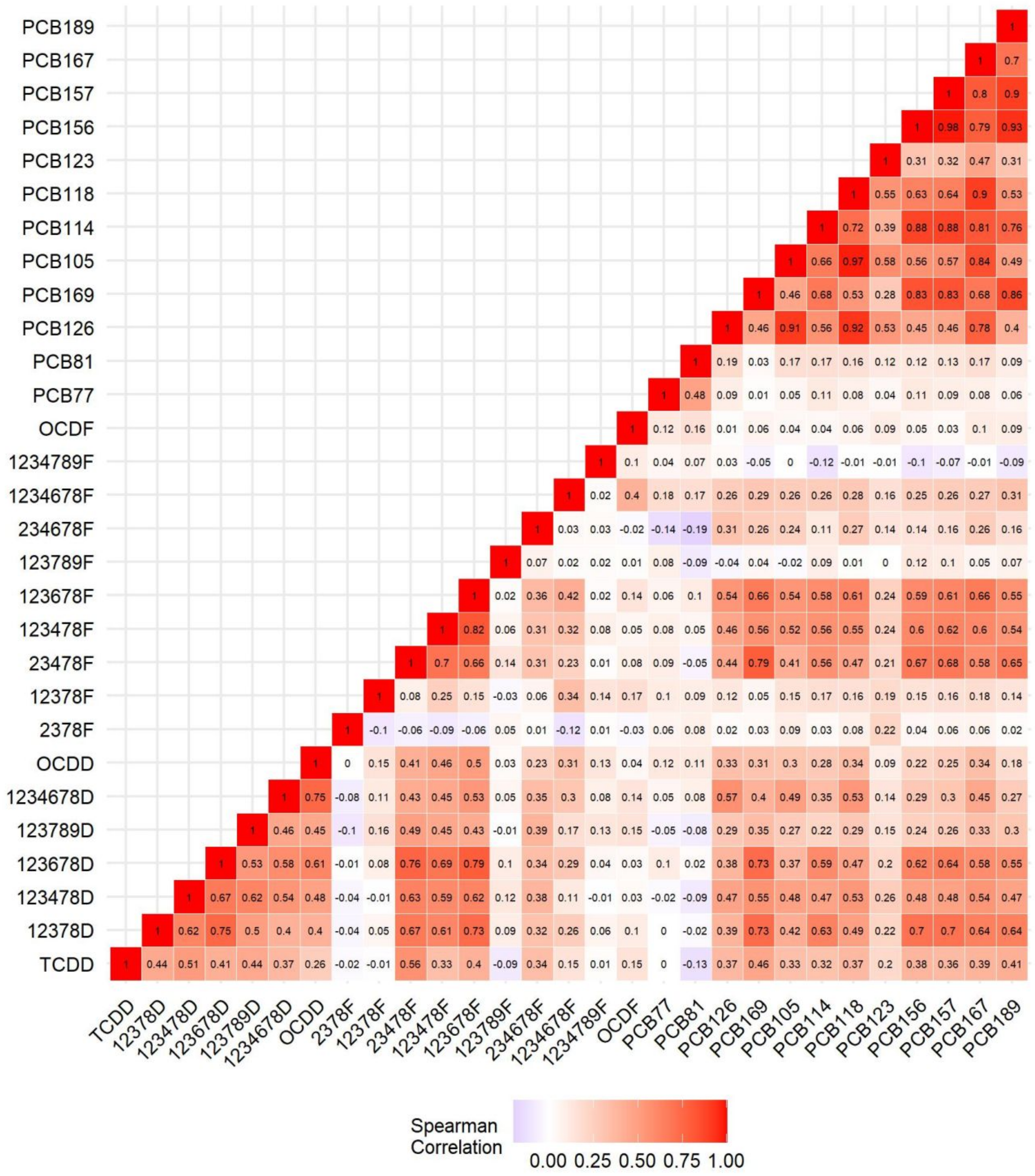

Figure S3. Correlation matrix of targeted dioxin(-like) compounds

Values in tiles were pairwise Spearman correlation coefficients among targeted dioxin(-like) compounds (n=29).

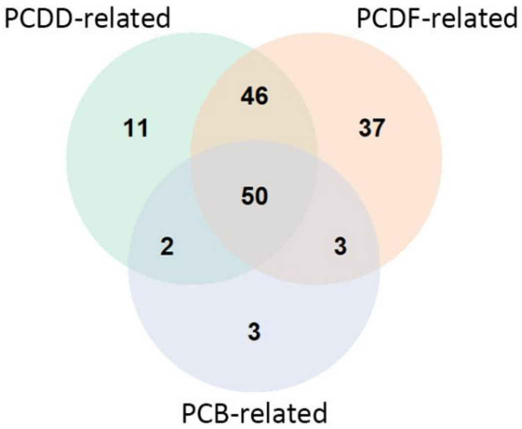

Figure S4. Venn diagram of PCDD-related, PCDF-related and PCB-related compounds

Dioxin(-like) related compounds were grouped by being related to PCDDs, PCDFs and PCBs. The respective numbers were 109, 136, 58.

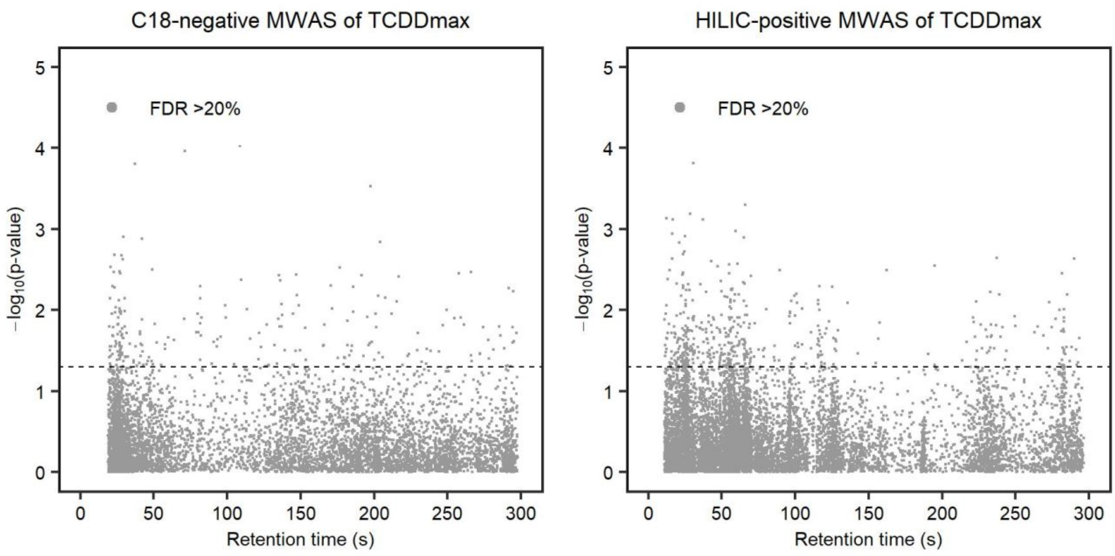

Figure S5. Manhattan plots of MWAS of maximum TCDD levels

Each dot represented a metabolic feature (in total 6,914 for C18-negative mode, 10,733 for HILIC-positive mode) and was presented by -$\log_{10}$(*p*-value), measuring the strength of association, against retention time of the feature. Dashed line represented raw *p*-value of 0.05. No feature was deemed significant under the threshold of false discovery rate 20%.

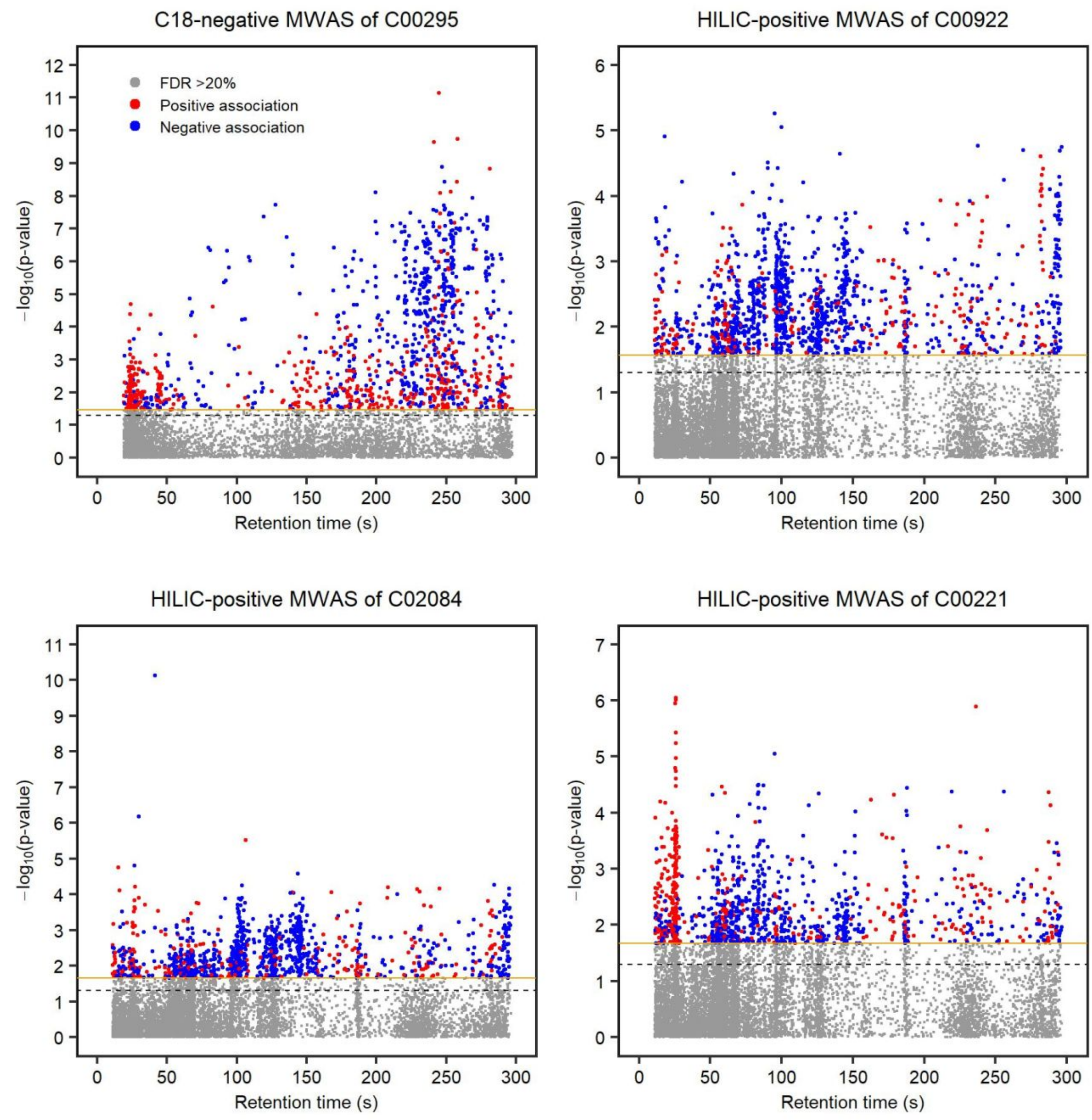

Figure S6. Manhattan plots of MWAS of certain dioxin(-like) related compounds

These four dioxin(-like) related compounds were significantly associated with >1000 metabolic features. Each dot represented a metabolic feature (in total 6,914 for C18-negative mode, 10,733 for HILIC-positive mode) and was presented by $-\log_{10}$(*p*-value), measuring the strength of association, against retention time of the feature. Significant features were colored according to the association direction. Dashed line represented raw *p*-value of 0.05; solid line represented FDR 20%.

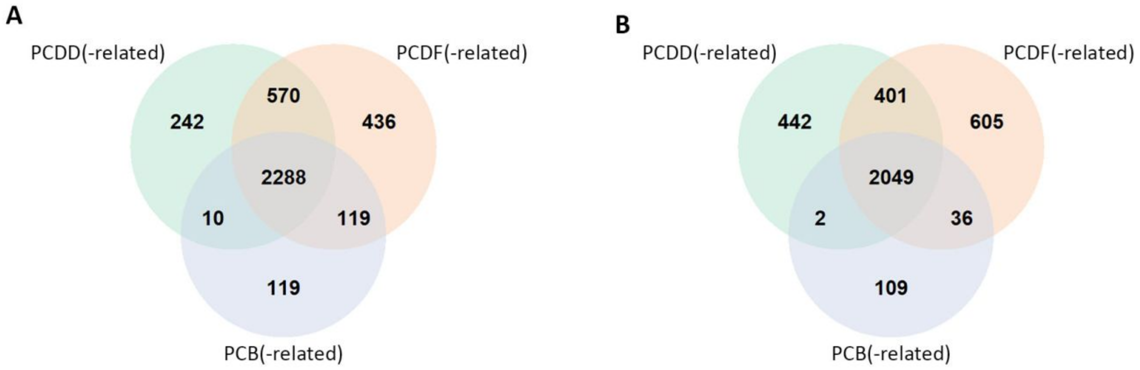

Figure S7. Venn diagram of metabolic features associated with PCDD(-related), PCDF(-related) and PCB(-related) compounds

C18-negative (A) and HILIC-positive (B) features were identified under FDR 20% of MWAS with at least one of known and related dioxin(-like) compounds. Metabolic features were categorized by being associated with PCDD(-related), PCDF(-related) and PCB(-related) compounds.

A.

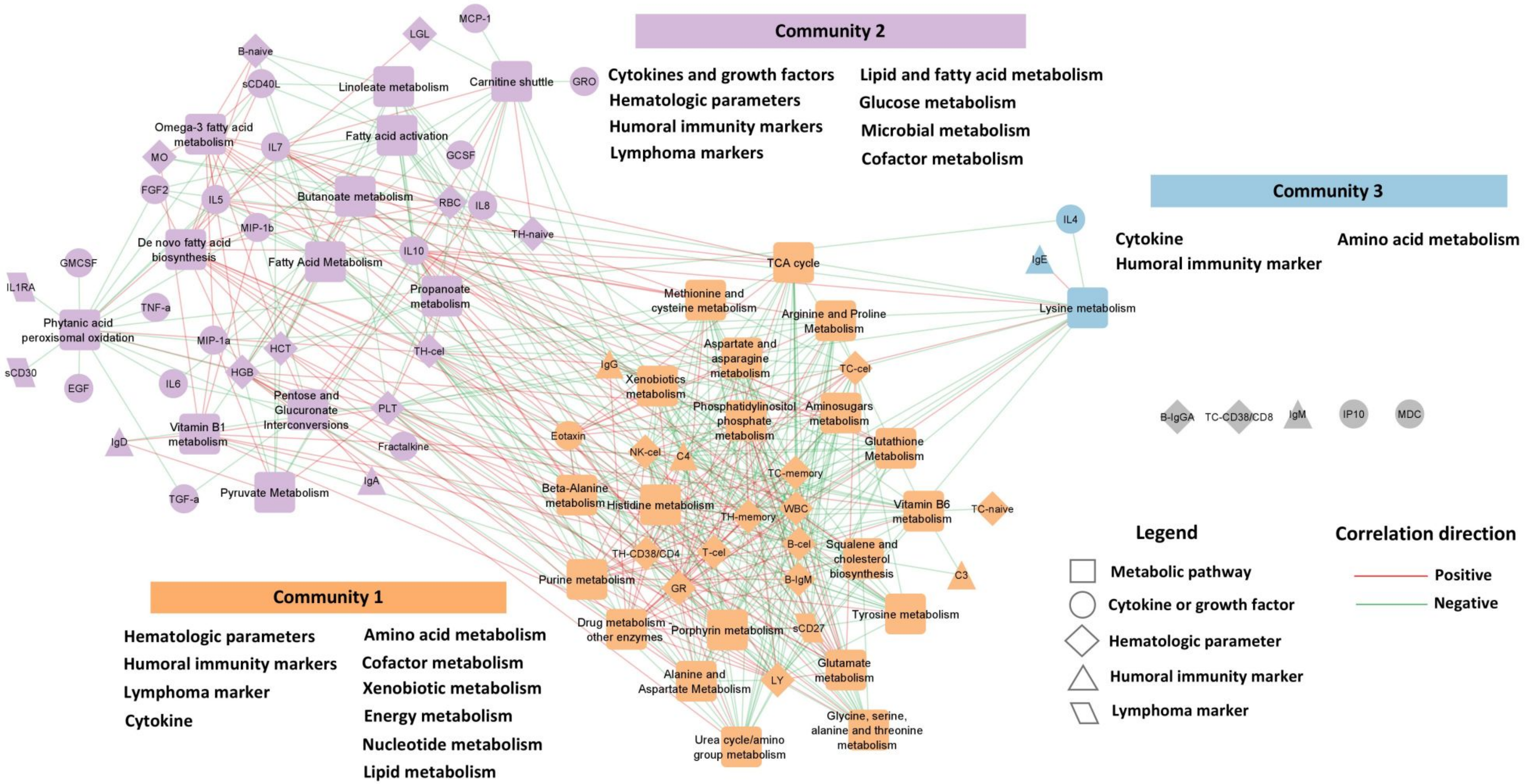

Community 2
Cytokines and growth factors
Hematologic parameters
Humoral immunity markers
Lymphoma markers
Lipid and fatty acid metabolism
Glucose metabolism
Microbial metabolism
Cofactor metabolism
Community 3
Cytokine
Humoral immunity marker
Amino acid metabolism
Community 1
Hematologic parameters
Humoral immunity markers
Lymphoma marker
Cytokine
Amino acid metabolism
Cofactor metabolism
Xenobiotic metabolism
Energy metabolism
Nucleotide metabolism
Lipid metabolism
Legend
Metabolic pathway
Cytokine or growth factor
Hematologic parameter
Humoral immunity marker
Lymphoma marker
Correlation direction
Positive
Negative
MCP-1
LGL
B-naive
sCD40L
Linoleate metabolism
Carnitine shuttle
GRO
Omega-3 fatty acid metabolism
IL7
Fatty acid activation
GCSF
MO
FGF2
IL5
Butanoate metabolism
RBC
IL8
MIP-1b
TH-naive
De novo fatty acid biosynthesis
IL10
GMCSF
Fatty Acid Metabolism
IL1RA
Propanoate metabolism
TNF-a
Phytanic acid peroxisomal oxidation
MIP-1a
HCT
TH-cel
sCD30
HGB
EGF
IL6
Pentose and Glucuronate Interconversions
PLT
Vitamin B1 metabolism
IgD
Fractalkine
IgA
TGF-a
Pyruvate Metabolism
TCA cycle
IL4
IgE
Lysine metabolism
Methionine and cysteine metabolism
Arginine and Proline Metabolism
Aspartate and asparagine metabolism
IgG
TC-cel
Xenobiotics metabolism
Phosphatidylinositol phosphate metabolism
Aminosugars metabolism
Eotaxin
Glutathione Metabolism
NK-cel
C4
TC-memory
Beta-Alanine metabolism
Histidine metabolism
TH-memory
WBC
Vitamin B6 metabolism
TC-naive
B-cel
Squalene and cholesterol biosynthesis
TH-CD38/CD4
T-cel
C3
Purine metabolism
GR
B-IgM
Tyrosine metabolism
Drug metabolism - other enzymes
Porphyrin metabolism
sCD27
Glutamate metabolism
Alanine and Aspartate Metabolism
LY
Glycine, serine, alanine and threonine metabolism
Urea cycle/amino group metabolism
B-IgGA
TC-CD38/CD8
IgM
IP10
MDC

B.

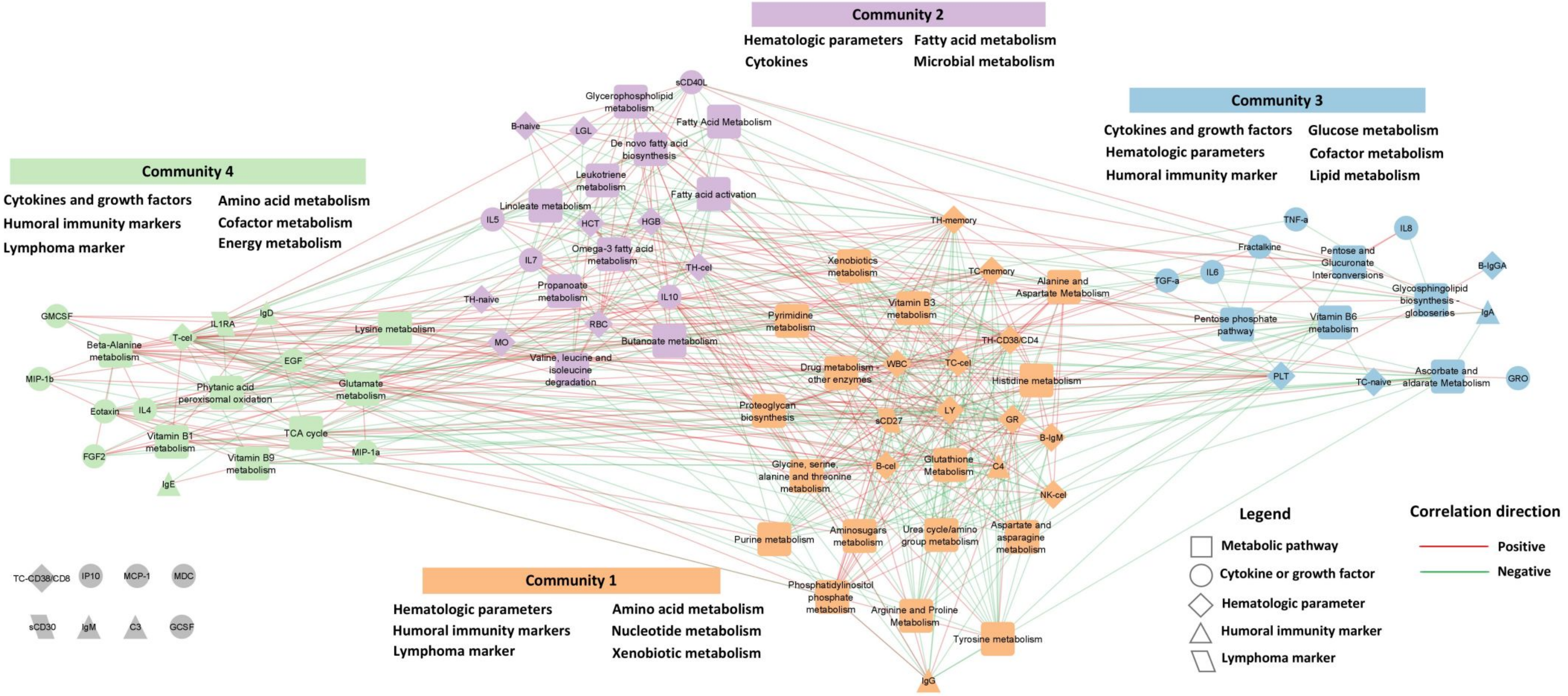

Community 2
Hematologic parameters
Fatty acid metabolism
Cytokines
Microbial metabolism
Community 3
Cytokines and growth factors
Glucose metabolism
Hematologic parameters
Cofactor metabolism
Humoral immunity marker
Lipid metabolism
Community 4
Cytokines and growth factors
Amino acid metabolism
Humoral immunity markers
Cofactor metabolism
Lymphoma marker
Energy metabolism
Community 1
Hematologic parameters
Amino acid metabolism
Humoral immunity markers
Nucleotide metabolism
Lymphoma marker
Xenobiotic metabolism
sCD40L
Glycerophospholipid metabolism
Fatty Acid Metabolism
B-naive
LGL
De novo fatty acid biosynthesis
Leukotriene metabolism
Fatty acid activation
Linoleate metabolism
IL5
HCT
HGB
TH-memory
Omega-3 fatty acid metabolism
IL7
TH-cel
Xenobiotics metabolism
TC-memory
Alanine and Aspartate Metabolism
Propanoate metabolism
IL10
TH-naive
Vitamin B3 metabolism
Pyrimidine metabolism
RBC
MO
Butanoate metabolism
TH-CD38/CD4
Valine, leucine and isoleucine degradation
Drug metabolism - other enzymes
WBC
TC-cel
Histidine metabolism
Proteoglycan biosynthesis
LY
sCD27
GR
B-IgM
Glycine, serine, alanine and threonine metabolism
B-cel
Glutathione Metabolism
C4
NK-cel
Purine metabolism
Aminosugars metabolism
Urea cycle/amino group metabolism
Aspartate and asparagine metabolism
Phosphatidylinositol phosphate metabolism
Arginine and Proline Metabolism
Tyrosine metabolism
IgG
TNF-a
IL8
Fractalkine
Pentose and Glucuronate Interconversions
B-IgGA
TGF-a
IL6
Glycosphingolipid biosynthesis - globoseries
Pentose phosphate pathway
Vitamin B6 metabolism
IgA
PLT
TC-naive
Ascorbate and aldarate Metabolism
GRO
GMCSF
IL1RA
IgD
T-cel
Lysine metabolism
Beta-Alanine metabolism
EGF
MIP-1b
Phytanic acid peroxisomal oxidation
Glutamate metabolism
Eotaxin
IL4
Vitamin B1 metabolism
TCA cycle
FGF2
Vitamin B9 metabolism
MIP-1a
IgE
TC-CD38/CD8
IP10
MCP-1
MDC
sCD30
IgM
C3
GCSF
Legend
Metabolic pathway
Cytokine or growth factor
Hematologic parameter
Humoral immunity marker
Lymphoma marker
Correlation direction
Positive
Negative

C.

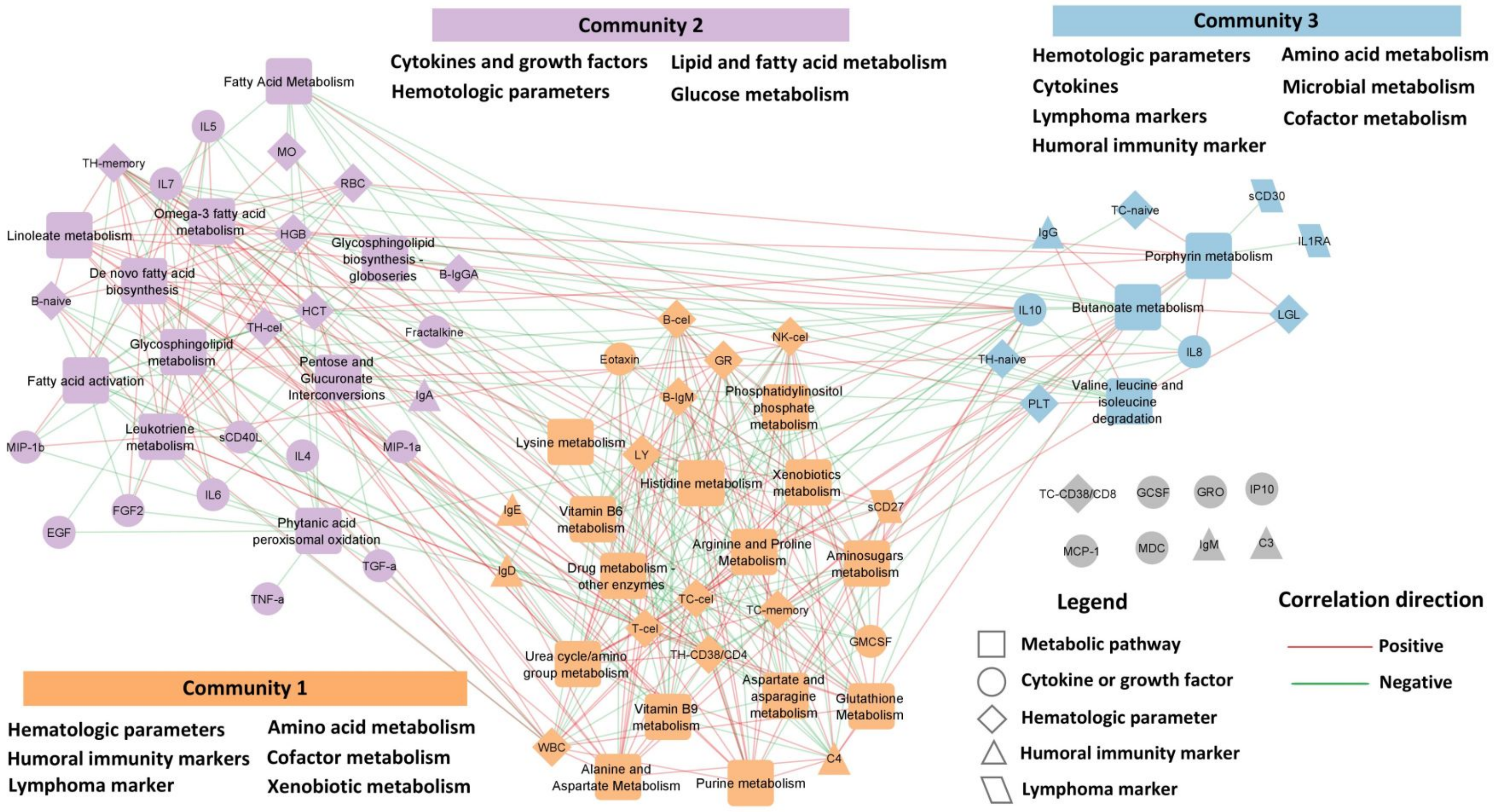

Figure S8. Network analysis of dioxin(-like) related pathways with immune markers, separately by PCDD(-related) (A), PCDF(related) (B), and PCB(-related) compounds (C)

Abbreviation: IL, interleukin; GMCSF, granulocyte-macrophage colony-stimulating factor; GCSF, granulocyte colony-stimulating factor; TNF-α, tumor necrosis factor alpha; EGF, epidermal growth factor; FGF2, fibroblast growth factor 2; GRO, melanoma growth stimulatory activity/growth-related oncogene; IP10,

interferon gamma-induced protein 10; MCP-1, monocyte chemotactic protein-1; MDC, macrophage derived chemokine; MIP-1α, macrophage inflammatory protein-1 alpha; MIP-1β, macrophage inflammatory protein-1 beta; sCD40L, soluble CD40 ligand; TGF-α, transforming growth factor alpha; sCD30, soluble CD30; sCD27, soluble CD27; IL1RA, interleukin 1 receptor antagonist; RBC, red blood cells; HGB, hemoglobin; HCT, hematocrit; PLT, platelet counts; MO, monocytes; GR, granulocytes; LY, lymphocytes; B-cel, B cells; B-naïve, naïve B cells; B-IgM, IgM+ memory B cells; B-IgG, IgG/IgA+ memory B cells; T-cel, T cells; TH-cell, T helper cells; TH-CD38/CD4, CD38/CD4 cells; TH-naïve, naïve CD4 cells; TH-memory, memory CD4 cells; TC-cel, cytotoxic T cells; TC-CD38/CD8, CD38/CD8 cells; TC-naïve, naïve CD8 cells; TC-memory, memory CD8 cells; LGL, large granular lymphocytes; NK-cel, natural killer cells.

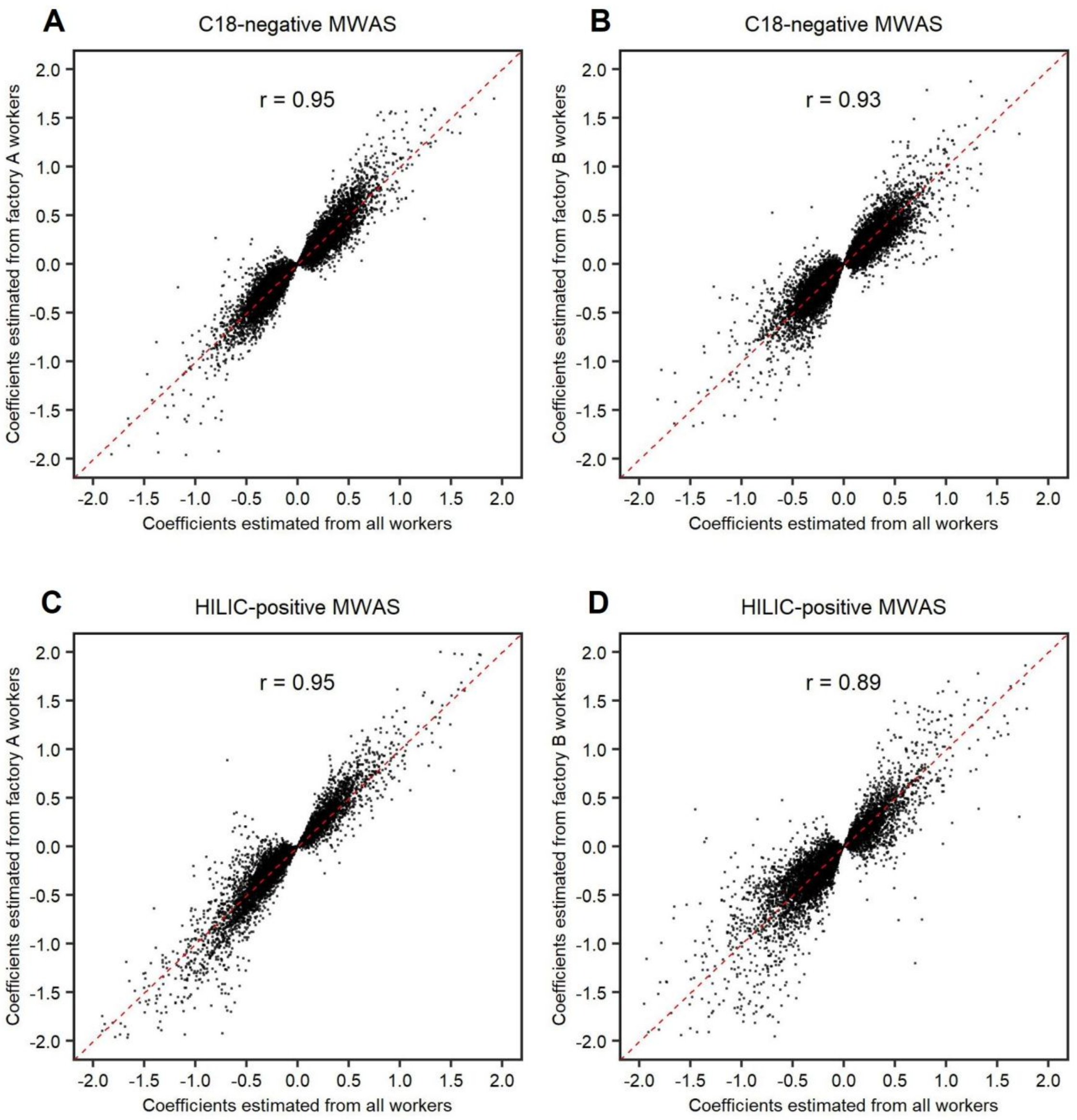

Figure S9. Comparison of main analyses and subgroup analyses on factory

Each dot represented one significant association (one targeted/related dioxin(-like) compound – one metabolic feature) detected in the main analysis of all workers (in total 22,557 C18-negative MWAS, 11,642 from HILIC-positive MWAS). They were displayed by coefficients from the main analysis against the corresponding values from subgroup analysis of factory A or factory B workers (adjusted by age and BMI). The dotted line is the line of identity.
